# The AXL-PYK2-PKCα axis as a nexus of stemness circuits in TNBC

**DOI:** 10.26508/lsa.202000985

**Published:** 2021-03-30

**Authors:** Lohit Khera, Yaron Vinik, Flavio Maina, Sima Lev

**Affiliations:** 1 Molecular Cell Biology Department, Weizmann Institute of Science, Rehovot, Israel; 2 Aix Marseille University, Le Centre National de la Recherche Scientifique (CNRS), Developmental Biology Institute of Marseille (IBDM) Unité Mixte de Recherche (UMR) 7288, Marseille, France

## Abstract

Cancer stem cells (CSCs) are implicated in tumor initiation, metastasis and drug resistance, and considered as attractive targets for cancer therapy. Here we identified a clinically relevant signaling nexus mediated by AXL receptor, PYK2 and PKCα and show its impact on stemness in TNBC. AXL, PYK2, and PKCα expression correlates with stemness signature in basal-like breast cancer patients, and their depletion in multiple mesenchymal TNBC cell lines markedly reduced the number of mammosphere-forming cells and cells harboring CSCs characteristic markers. Knockdown of PYK2 reduced the levels of AXL, PKCα, FRA1, and PYK2 proteins, and similar trend was obtained upon PKCα depletion. PYK2 depletion decreased AXL transcription through feedback loops mediated by FRA1 and TAZ, whereas PKCα inhibition induced redistribution of AXL to endosomal/lysosomal compartment and enhanced its degradation. PYK2 and PKCα cooperate at a convergence point of multiple stemness-inducing pathways to regulate AXL levels and concomitantly the levels/activation of STAT3, TAZ, FRA1, and SMAD3 as well as the pluripotent transcription factors Nanog and Oct4. Induction of stemness in TNBC sensitized cells to PYK2 and PKCα inhibition suggesting that targeting the AXL-PYK2-PKCα circuit could be an efficient strategy to eliminate CSCs in TNBC.

## Abstract (precis)

A clinically relevant AXL-PYK2-PKCα axis where PYK2 and PKCα act as signaling nodes and functionally cooperate to converge stemness promoting pathways and regulate Oct4 and Nanog pluripotent TFs.

## Introduction

Cancer stem cells (CSCs) represent a small fraction of cancer cells, characterized by specific cellular markers, self-renewal and tumor-initiating capacities (1, 2). This subpopulation of cells, also known as tumor-initiating cells (TICs), are commonly associated with drug-resistance and metastatic potential, and are considered as promising targets for therapeutic intervention.

Breast cancer (BC)-initiating cells typically express high levels of CD44, a surface receptor for the extracellular matrix protein Hyaluronan, low levels of CD24, and exhibit high aldehyde dehydrogenase (ALDH) activity (3). Other cell surface proteins, including CD326 (EpCAM), epithelial specific antigen, CD133, CD166, CD47, CD201, and ABCG2 have been reported as CSC markers for different BC subtypes and drug-resistant tumors (4, 5). CD44^+^/CD24^−^ ratio is particularly enriched in CSCs of triple negative breast cancer (TNBC) (6), a highly aggressive BC subtype defined by the absence of estrogen and progesterone hormone receptors, and of HER2 amplification (7).

Multiple studies have shown that CSCs have the capacity to regenerate bulk tumors that are mostly composed of non-cancer stem cells (NCSCs) (8), whereas NCSCs can dedifferentiate and acquire a CSC phenotype upon epithelial mesenchymal transition (EMT) (9). EMT is a multistep process involved in cancer metastasis, mediated by activation of Slug/Snail, Twist and/or Zeb transcription factors (TFs), and characterized by a specific gene signature. Importantly, EMT and BC initiating cells display very similar gene signatures and phenotypic properties (9, 10).

Previously we showed that the non-receptor tyrosine kinase PYK2 positively regulates EMT in TNBC (11). Similarly, the tyrosine kinase receptor AXL of the TAM (Tyro-Axl-Mer) family was reported to be essential for EMT in BC and to regulate CSC self-renewal and chemoresistance (12, 13, 14). AXL up-regulation in several human cancers including TNBC (15) is frequently associated with resistance to different chemotherapeutic agents and targeted therapies, including MEK (16), PI3K/AKT, BET, and EGFR (17). These observations underscore the potential clinical benefit of AXL inhibition/down-regulation in drug-resistant tumors, which commonly display EMT properties and are enriched in CSCs (18).

Numerous studies have highlighted the role of different signaling pathways and signaling intermediates in regulating BC stem cells (BCSCs) self-renewal and maintenance of stemness properties, including the TGFβ, IL6/IL8, Hedgehog, Notch, Wnt, AXL, and the Hippo pathways (6). TGFβ, a potent EMT inducer in mammary cells, enhances stemness of chemotherapy-resistant TNBC cells (19). Activation of STAT3 signaling by different growth factors and cytokines including IL6, has been implicated in EMT, self-renewal of BCSCs as well as acquisition of stemness properties of doxorubicin resistant TNBC cells (20, 21), whereas TAZ was reported to be essential for metastatic activity and chemoresistance of BCSCs (22). Different signaling components, including the gap junction protein connexin 26, FAK (23), CDK4 (24), JAK/STAT3 (25), BET proteins (26), and PKCα (27) among others, have been proposed as promising targets to eliminate CSCs in TNBC.

The numerous targets that have been reported to affect CSCs might be associated with the multiple stemness-inducing pathways in TNBC, their crosstalk and feedback regulation. Currently, however, none of these targets have a proven clinical efficacy as a mono-therapeutic agent. Hence, targeting of signaling nodes that function at a convergent point of different stemness-promoting pathways could be an effective approach to eliminate CSCs in TNBC.

Here we identified two cytosolic kinases; PYK2 and PKCα as key signaling nodes of different stemness pathways in TNBC and show their clinical relevance for stemness signature in basal-like (BL) patients, and their pleotropic effects on critical TFs that regulate stem-like properties. Furthermore, we show that PYK2 depletion and/or inhibition of its kinase activity markedly reduced the steady-state levels of AXL receptor, PKCα and FRA1 TF in various mesenchymal/mesenchymal stem–like (M/MSL) TNBC cell lines. We found that the AXL-PYK2-PKCα circuit regulates stemness in TNBC through crosstalk with other stemness pathways and feedback regulatory loops. Consistent with these results, we show that TGFβ enhanced stemness properties and concurrently sensitized TNBC to PKCα and PYK2 inhibition, implying that co-inhibition of these two kinases could preferentially target CSCs in TNBC.

## Results

### Interplay between AXL, PYK2, and PKCα in TNBC

**Figure 1.**
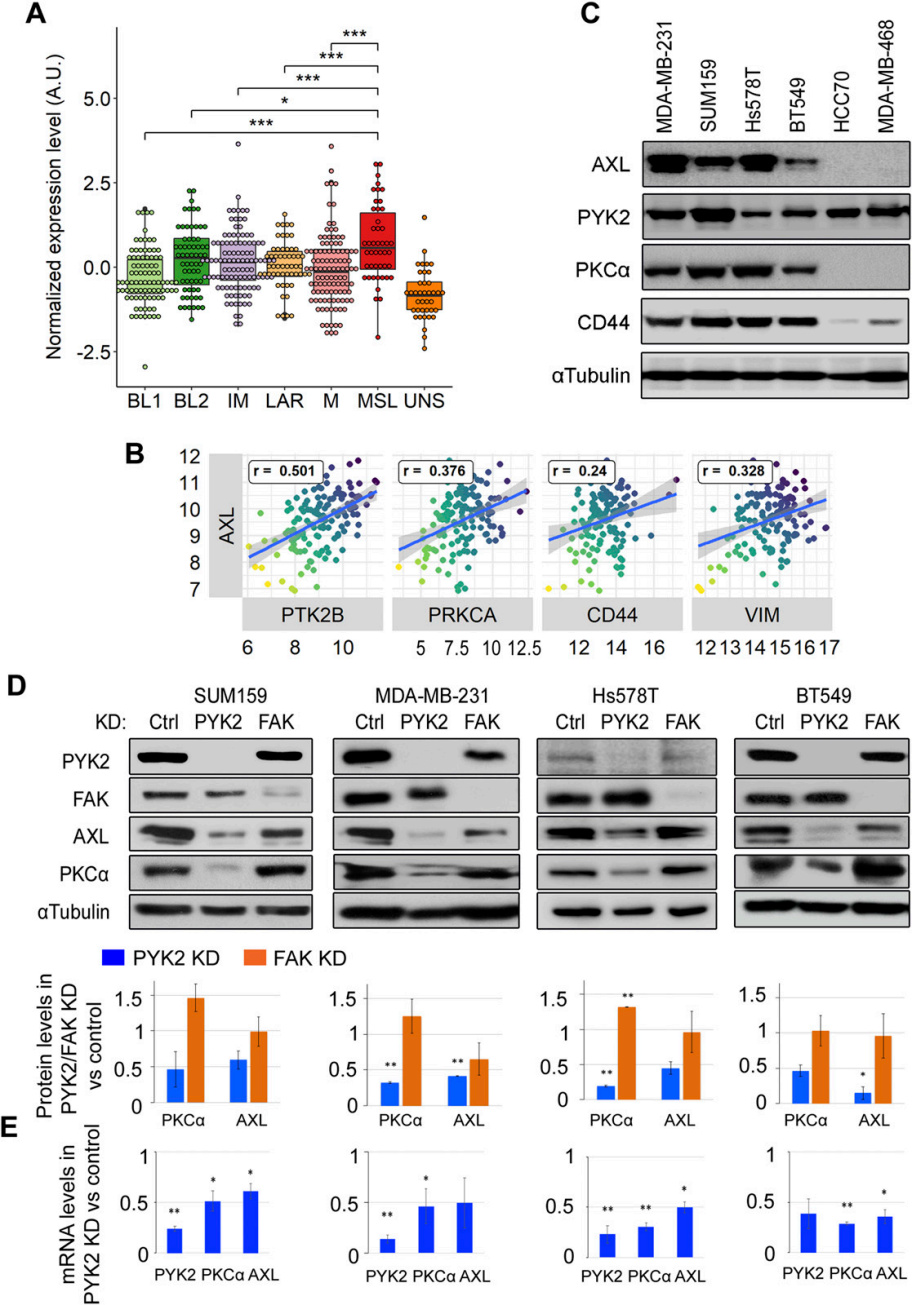
AXL expression correlates with PYK2 and PKCα in triple negative breast cancer (TNBC) and is influenced by PYK2 expression. **(A)** Expression of AXL in TNBC patients from 19 datasets (taken from reference 28). The expression level was normalized for each dataset. TNBC subtypes: BL1 and BL2, basal-like 1 and 2; IM, immunomodulatory; LAR, luminal androgen-receptor positive; M, mesenchymal; MSL, mesenchymal stem–like; UNS, unspecified. **(B)** Pearson’s correlation between the expression of AXL and other genes in The Cancer Genome Atlas BL breast cancer patients (n = 142 patients). *PRKCA* encodes PKCα, *PTK2B* encodes PYK2, *VIM* encodes Vimentin. **(C)** Western blot analysis of AXL, PYK2, PKCα, and CD44 in four M/MSL (MDA-MB-231, SUM159, Hs578T, and BT549) and two BL (HCC70, MDA-MB-468) TNBC cell lines. **(D)** Western blot analysis of the indicated proteins in control, PYK2 knockdown (KD), or FAK KD TNBC (M/MSL) cell lines. The protein levels were estimated by densitometric analysis (ImageJ) and mean values of two experiments are shown in the bar graph as fold of control ± SD. **(E)** Bar graphs showing qRT-PCR analysis of PYK2, PKCα, and AXL transcripts in PYK2 KD cells versus control. Mean values ± SD of three experiments each for SUM159 and MDA-MB-231 cells, and two experiments each for Hs578T and BT549 are shown. *P*-values of PYK2 KD versus control (*t* test) * < 0.05; ** < 0.01; *** < 0.001.

**Figure S1.**
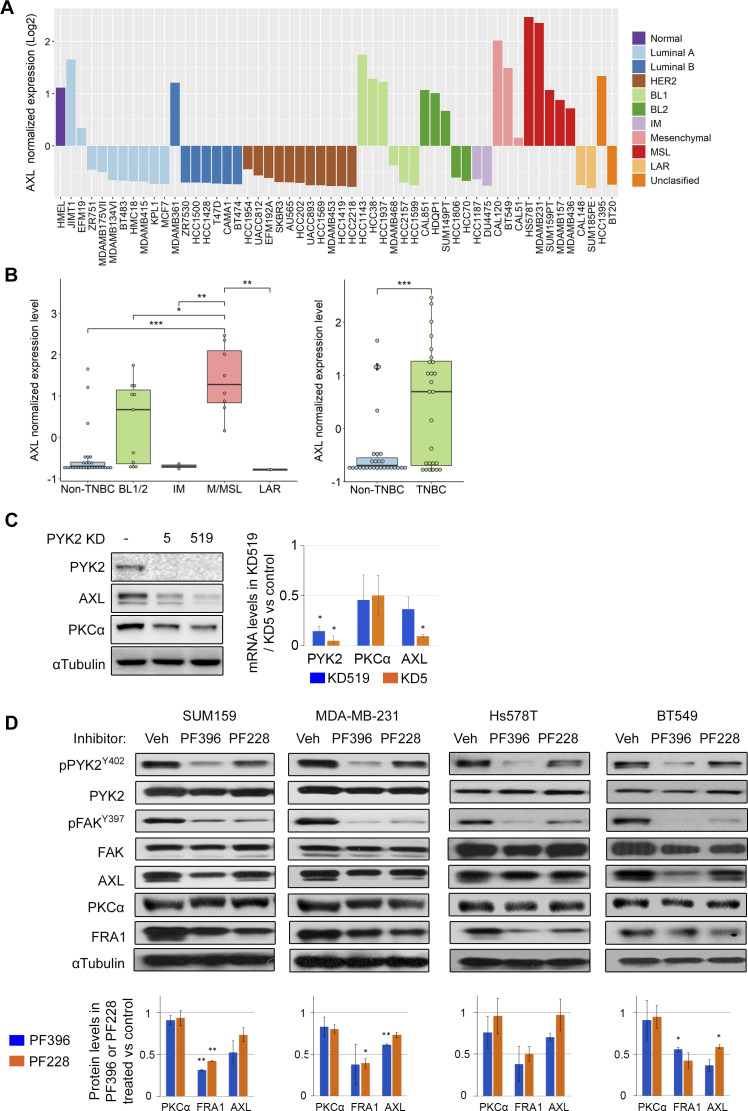
Expression of AXL in breast cancer (BC) cell lines and effect of PYK2/FAK inhibition on AXL, PKCα, and FRA1 levels. **(A, B)** Normalized expression of AXL in BC cell lines. Data are taken from cancer cell line encyclopedia dataset. BC cell lines subtypes: BL1 and 2, basal-like 1 and 2, IM, immunomodulatory, MSL, mesenchymal stem–like. Luminal A, B, and HER2-positive BC cell lines constitute the non-triple negative breast cancer (TNBC) group, whereas BL1, BL2, IM, M (mesenchymal), MSL, LAR, and unclassified constitute the TNBC group. **(C)** Knockdown of PYK2 in SUM519 cells by two shRNAs (# 5, # 519) similarly affects PYK2, PKC, AXL protein levels, and transcripts, as determined by Western blot and qRT-PCR analysis, respectively. Bar graphs showing RT-qPCR analysis of PYK2, PKCα, and AXL transcripts in indicated PYK2 KD cells versus control. Mean values ± SD of two experiments are shown. *P*-values of PYK2 KD versus control (*t* test) * < 0.05; ** < 0.01. **(D)** The indicated M/MSL TNBC cell lines were treated overnight either with FAK inhibitor (PF228, 10 μM) or with FAK/PYK2 dual inhibitor (PF396, 5 μM). Controls (Veh) were treated with DMSO. The levels of the indicated proteins or their phosphorylation were assessed by Western blot. Quantification of protein bands intensities from two experiments is shown in the bottom. Mean values ± SD of two experiments are shown. *P*-values of inhibitor treatment versus control (*t* test) * < 0.05; ** < 0.01.

Previous studies have shown that AXL expression is strongly induced during EMT, drug resistance and metastasis in BC (13, 17). Expression profiling analysis revealed its high enrichment in MSL subtype of TNBC patients (28) (Fig 1A). Further analysis of TNBC patients from The Cancer Genome Atlas (TCGA) dataset showed high correlation between *AXL* and *PTK2B* (PYK2 gene) expression (r = 0.5, *P* < 0.001), *AXL* and *PRKCA* (PKCα gene) expression (r = 0.37, *P* < 0.001), and *AXL* and the EMT markers, *CD44* (r = 0.24, *P* < 0.01) and *vimentin* (0.328, *P* < 0.01) (Fig 1B). High levels of AXL transcript were also observed in M/MSL TNBC cell lines of the Cancer Cell Line Encyclopedia (CCLE) (Fig S1A and B). Western blot (WB) analysis confirmed high level of AXL protein in M/MSL cell lines (MDA-MB-231, SUM159, Hs578T, and BT549) compared to basal-like (HCC70, MDA-MB-468) TNBC cell lines (Fig 1C). Similarly, PKCα, which was shown to be essential for BCSC formation (27), and the characteristic CSCs marker CD44 (4), were also enriched in M/MSL cell lines (Fig 1C).

We previously showed that PYK2 depletion markedly reduced the level of CD44 in TNBC cells (11), and further analysis of at least four different M/MSL TNBC cell lines showed profound effects of PYK2 knockdown (KD) on the steady-state levels of AXL and PKCα (Figs 1D and S1C). However, depletion of FAK, a closely related kinase of PYK2 (29), had marginal effects on the protein levels of PKCα or AXL, and similar effects were obtained with the FAK specific kinase inhibitor PF228 (Fig S1D). The dual PYK2/FAK kinase inhibitor (PF396) reduced the steady-state level of AXL but had no effect on the steady-state levels of PKCα (Fig S1D), thus highlighting the specific influence of PYK2.

**Figure S2.**
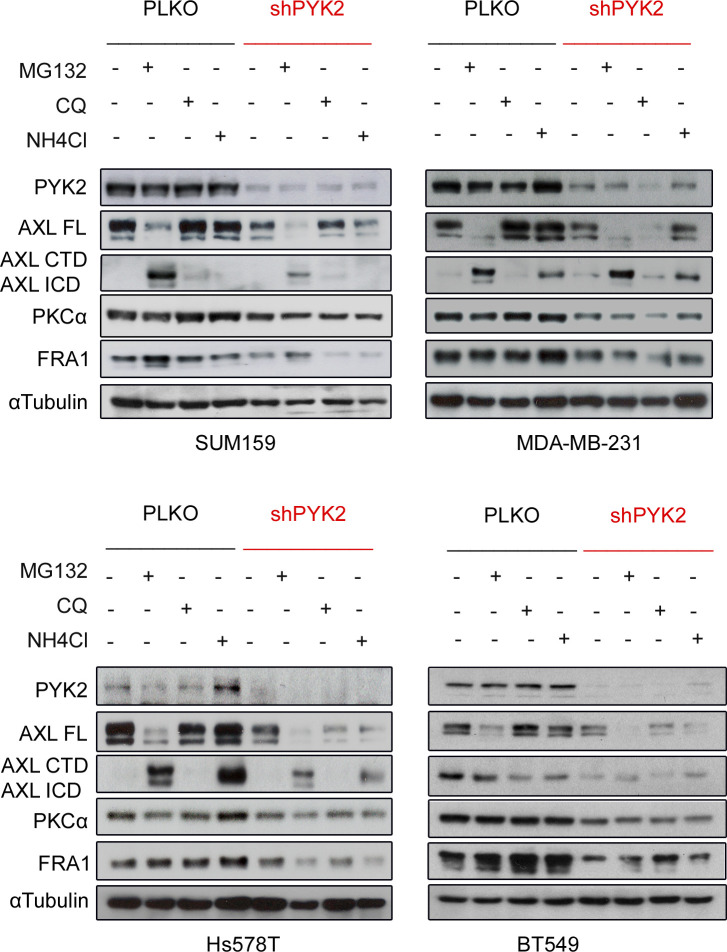
Inhibition of protein degradation pathways could not rescue PYK2 KD effect on AXL-PKCα-FRA1. The indicated mesenchymal/mesenchymal stem–like cell lines with control and PYK2 KD were treated with the proteasomal inhibitor MG132 (10 μM) or with the lysosomal inhibitors Chloroquine (CQ; 50 μM) or NH_4_Cl (10 mM) for 4 h. Expression levels of the indicated proteins was assessed by Western blot. MG132 treatment, enhanced AXL cleavage as previously reported (64). AXL FL (Full length) AXL ICD (Intra-cellular domain), AXL CTD (C-terminal domain). Source data are available for this figure.

The substantial effects of PYK2 KD on AXL and PKCα levels in multiple M/MSL TNBC lines led us to examine its influence on their transcripts using qRT-PCR. As shown in Fig 1E, PYK2 KD reduced the transcript levels of PKCα and AXL (by ∼50–70%) in the four representative M/MSL cell lines; SUM-159, MDA-MB-231, Hs578T, and BT-549 (will be used throughout this study). The proteasomal inhibitor MG132 as well as the lysosomal inhibitors Chloroquine and NH_4_Cl could not restore the protein levels of AXL or PKCα in PYK2 KD cells (Fig S2). Collectively, these results indicate that PYK2 depletion markedly affects the transcript and protein levels of AXL and PKCα in M/MSL TNBC cell lines.

### FRA1 is regulated by PYK2 and PKCα and affects AXL and PKCα levels

**Figure 2.**
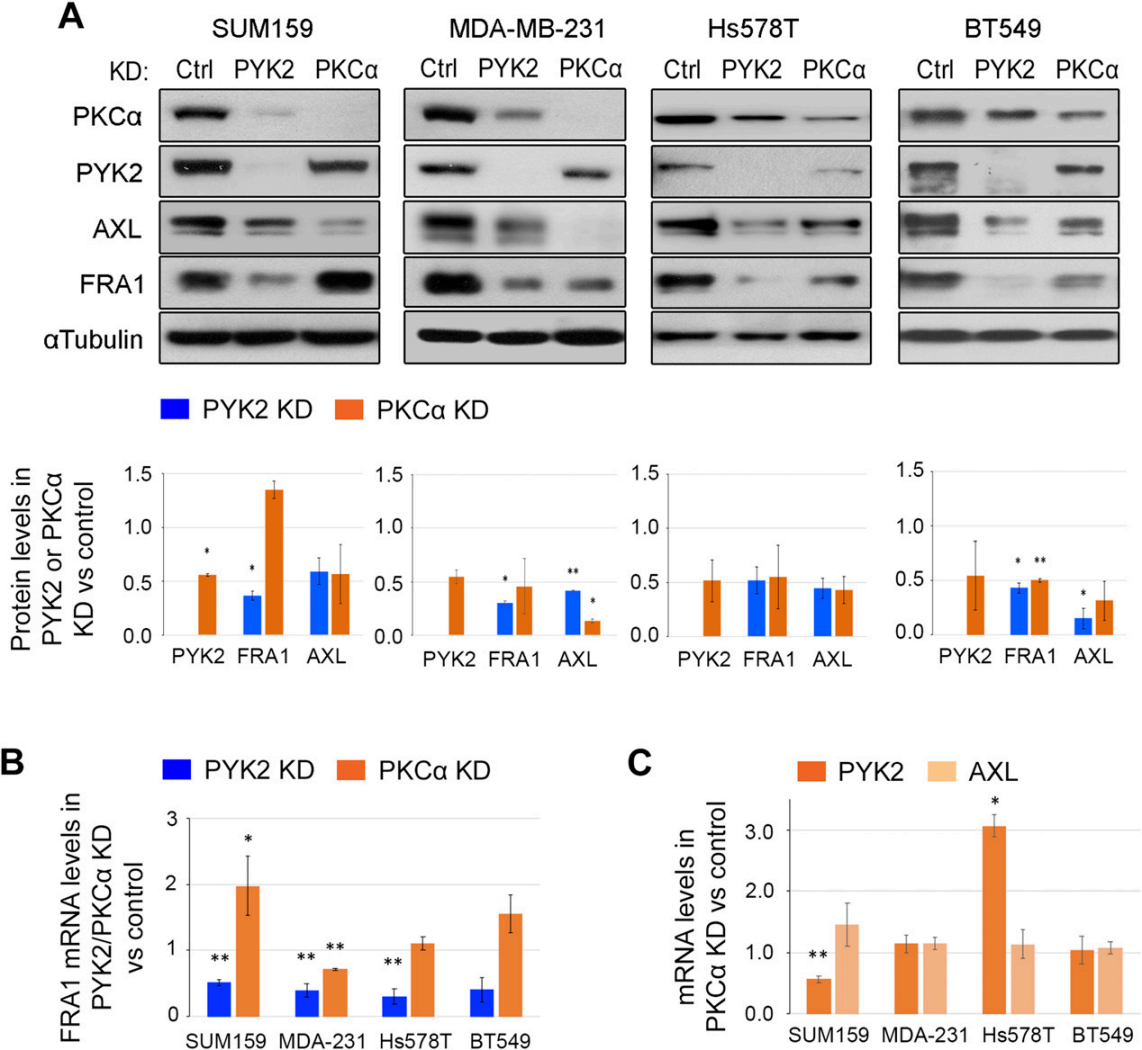
PYK2 and PKCα distinctly affect the AXL-PYK2-PKCα axis. **(A)** Western blot analysis of the indicated proteins in control, PYK2 KD or PKCα KD triple negative breast cancer (mesenchymal/mesenchymal stem–like) cell lines. The protein levels were estimated by densitometric analysis (ImageJ) and mean values of two experiments are shown in the bar graph as fold of control. **(B)** Bar graphs showing qRT-PCR analysis of FRA1 transcript in PYK2 or PKCα KD cells compared with control. Mean values ± SD of two to three experiments are shown. **(C)** Bar graphs showing qRT-PCR analysis of PYK2 and AXL transcripts in PKCα KD cells compared with control. Mean values ± SD of three experiments each for SUM159, MDA-MB-231, and Hs578T, and two experiments for BT549 are shown. *P*-values of PKCα KD versus control (*t* test) *< 0.05; ** < 0.01.

**Figure S3.**
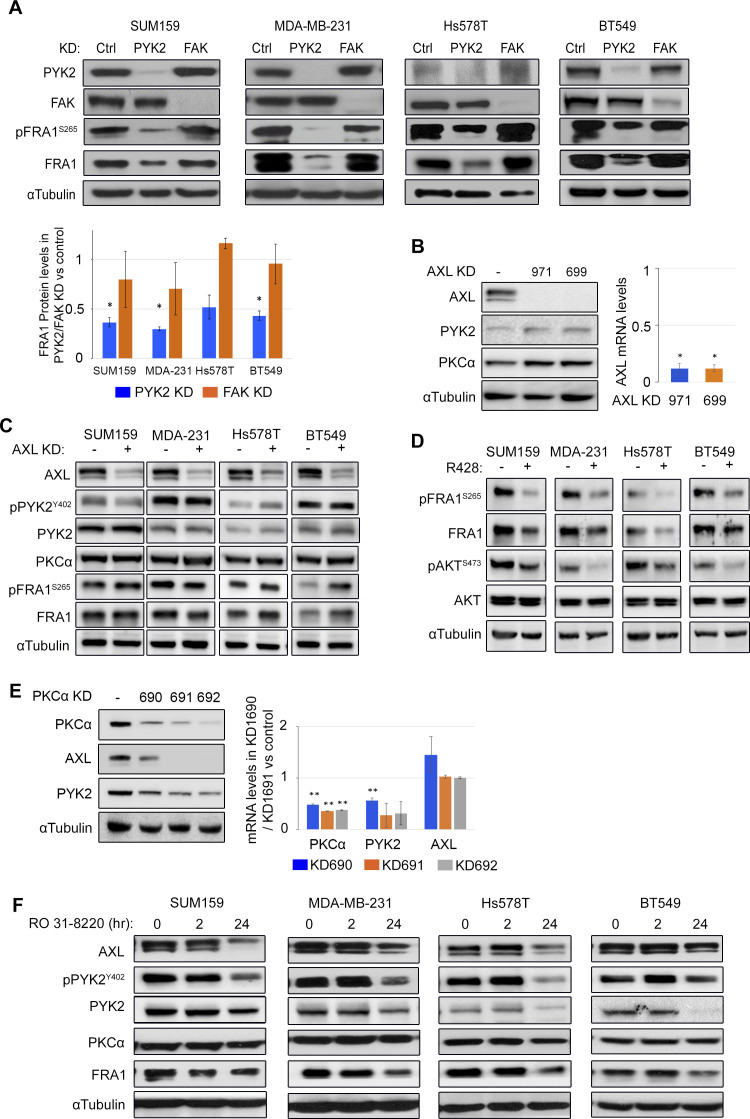
Effects of knockdown/inhibition of PYK2, PKCα, and AXL on the AXL-PYK2-PKCα-FRA1 axis. **(A)** The level of total and phospho-FRA1(S265) was assessed in the control, PYK2 KD or FAK KD mesenchymal/mesenchymal stem–like (M/MSL) triple negative breast cancer cell lines by Western blot (WB). Bar graphs on the bottom show mean values ±SD of results from two experiments. *P*-values of PYK2/FAK KD versus control (*t* test) * < 0.05. **(B)** Knockdown of AXL in MDA-MB-231 cells by two shRNAs (# 971, # 699) similarly affects PYK2, PKC, AXL protein levels, and transcripts, as determined by WB and qRT-PCR analysis, respectively. Bar graphs showing qRT-PCR analysis of AXL transcripts in indicated AXL KD cells versus control. Mean values ± SD of two experiments are shown. *P*-values of AXL KD versus control (*t* test) * < 0.05. **(C)** The effects of AXL KD on the expression and phosphorylation of the AXL-PYK2-PKCα-FRA1 axis in the indicated M/MSL triple negative breast cancer cell lines were assessed by WB. **(D)** The indicated M/MSL cell lines were treated with AXL kinase inhibitor R428 (2 μM) for 24 h and then analyzed by WB for phosphorylation of FRA1(S265) and AKT(S473). **(E)** Knockdown of PKCα in SUM159 cells by three shRNAs (# 690, # 691, # 692) similarly affects PYK2, PKC, AXL protein levels, and transcripts, as determined by WB and qRT-PCR analysis, respectively. Bar graphs showing qRT-PCR analysis of PKCα, PYK2, and AXL transcripts in indicated PKCα KD cells versus control. Mean values ± SD of two experiments are shown. *P*-values of PKCα KD versus control (*t* test) * < 0.05; ** < 0.01. **(F)** The indicated M/MSL cell lines were treated with PKC inhibitor RO-31-8220 for either 2 or 24 h (2 μM for SUM159 and BT549; 1 μM for Hs578T and MDA-MB-231 cells) and then analyzed by WB for components of the AXL-PYK2-PKCα-FRA1 axis.

The significant effects of PYK2 KD on AXL and PKCα transcripts led us to examine its influence on the level and activation of FRA1 TF. It was previously shown that AXL is a universal target of FRA1 (30, 31), and previous studies suggest that PKCα (27) and AXL are upstream activators of FRA1 (32). FRA1 levels were examined in PYK2-, PKCα-, or AXL-depleted M/MSL TNBC cell lines (the four representative M/MSL cell lines). As shown in Fig 2A, depletion of PYK2 substantially reduced the level of FRA1 and its phosphorylation (pS265) (Fig S3A) in all four cell lines. PKCα-deletion reduced the level of FRA1 protein in all the lines except SUM159 (Fig 2A), whereas AXL-depletion had no significant effects on either total PYK2, PKCα, or FRA1 (Fig S3B and C). Nevertheless, R428, an AXL kinase inhibitor, reduced FRA1 phosphorylation in the four cell lines (Fig S3D) as expected. Intriguingly, PKCα knockdown also reduced the protein levels of PYK2 and AXL to different extents (Figs 2A and S3E) and similar effects were obtained by the PKC inhibitor RO 31-8220 (Fig S3F). The mutual effects of PYK2 and PKCα on each other, and on the protein levels of AXL and FRA1, indicate a putative signaling circuit consisting AXL-PYK2-PKCα-FRA1.

**Figure 3.**
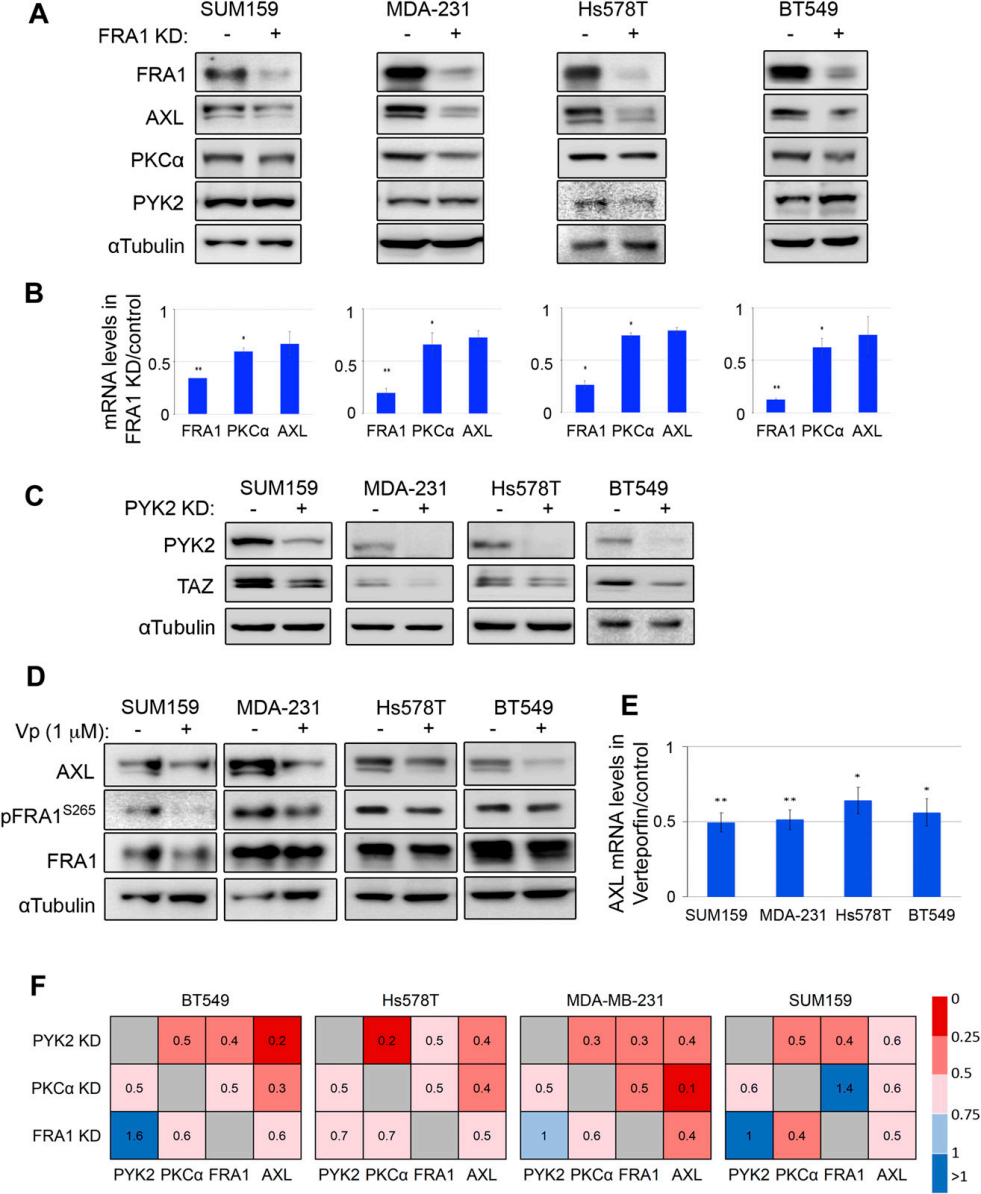
FRA1 and TAZ levels are influenced by PYK2 and affect AXL transcription. **(A)** Western blot (WB) analysis of the indicated proteins in the control and FRA1 KD triple negative breast cancer (mesenchymal/mesenchymal stem–like [M/MSL]) cell lines. **(B)** Bar graphs showing RT-qPCR analysis of FRA1, PKCα and AXL transcripts in FRA1 KD compared with control cells. Mean values ± SD of three experiments each for MDA-MB-231 and BT549, and two experiments each for SUM159 and Hs578T are shown. **(C)** WB analysis of TAZ protein in the indicated control and PYK2 KD cell lines. **(D, E)** The indicated triple negative breast cancer M/MSL cell lines were treated with 1 μM verteporfin (Vp) for 24 h. Controls were treated with equal volume of DMSO. WB (D) and qRT-PCR analysis (E) of AXL protein and transcript levels, respectively, are shown. Mean values ± SD of three experiments are shown. *P*-values of FRA1 KD or verteporfin treatment versus control (*t* test)* < 0.05; ** < 0.01. **(F)** Heat map of protein levels of the AXL-PYK2-PKCα-FRA1 axis in the four M/MSL cell lines, in PYK2-, PKCα-, or FRA1-KD cells. Mean values of two experiments were used to generate the heat map.

To better characterize this circuit, we assessed the impact of PYK2 or PKCα depletion on the transcription levels of FRA1 as well as of other components of the circuit by qRT-PCR. As shown, PKCα depletion had no significant effects on FRA1 or AXL transcripts; it slightly reduced the expression level of FRA1 in MDA-MB-231 and of PYK2 in SUM159 cells (Fig 2B and C), whereas KD of PYK2 markedly reduced the level of FRA1 (Fig 2B) concomitant with AXL and PKCα transcripts in the four cell lines (Fig 1E). These results suggest that PYK2 depletion affects AXL level through a feedback loop mediated by FRA1. To explore this possibility, we knocked down FRA1 in the four TNBC cell lines and assessed its influence on AXL, PKCα, and PYK2 proteins by WB. As shown in Fig 3A, FRA1 KD reduced the steady-state levels of AXL and PKCα in all the lines, and qRT-PCR analysis revealed ∼30–50% reduction in AXL and PKCα transcripts (Fig 3B). The partial effects of FRA1 KD on AXL transcript imply that an additional TF might be involved. Indeed, it was previously shown that AXL is regulated by the transcriptional co-activator TAZ (33), and we previously showed that PYK2 stabilizes TAZ in multiple TNBC cell lines (34), suggesting that the effects of PYK2 on AXL could be mediated by both FRA1 and TAZ. Consistent with this hypothesis, we found that PYK2 depletion indeed reduced TAZ levels in all four M/MSL lines (Fig 3C), and that TAZ inhibitor verteporfin reduced AXL protein (Fig 3D) and mRNA levels (Fig 3E). Collectively, our data show the interdependency between the proteins of the AXL-PYK2-PKCα-FRA1 circuit as summarized in Fig 3F.

### PKC inhibition modulates AXL trafficking and lysosomal degradation of AXL and PYK2

**Figure S4.**
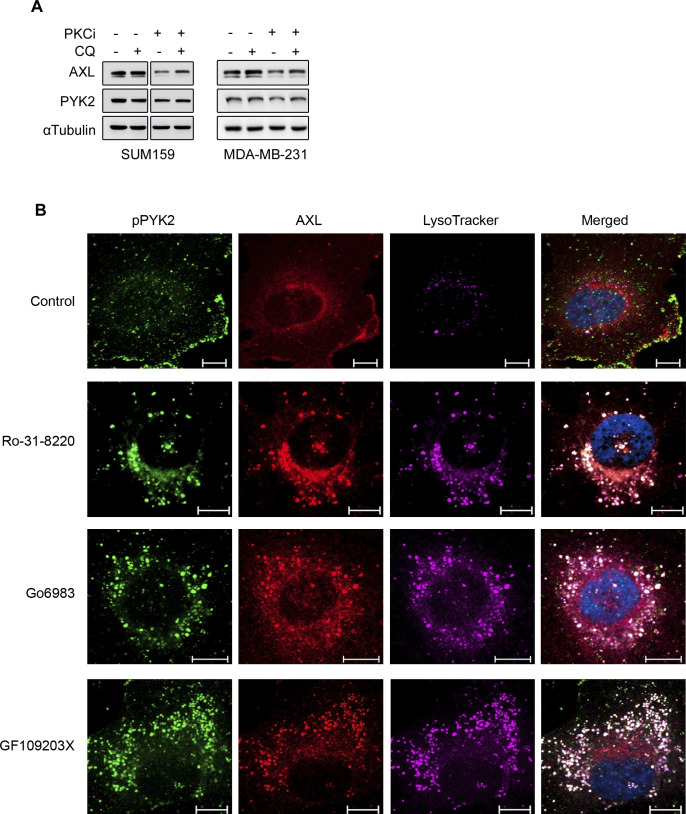
PKC inhibitors induce redistribution of pPYK2 and AXL. **(A)** SUM159 and MDA-MB-231 cells were treated with RO-31-8220 for 24 h, followed by 3 h treatment with 50 μM chloroquine (CQ). Western blot analysis was performed to assess levels of PYK2 and AXL. **(B)** SUM159 cells were treated with 2 μM of the indicated PKC inhibitors (RO-31-8220, Gö6983, or GF109203X) for 2 h. The cells were fixed and immune-stained for pPYK2^Y402^ and AXL. Shown are confocal images of pPYK2^Y402^ and AXL, and lysotracker subcellular localization along with the merged image. Scale bar, 10 μm.

**Figure 4.**
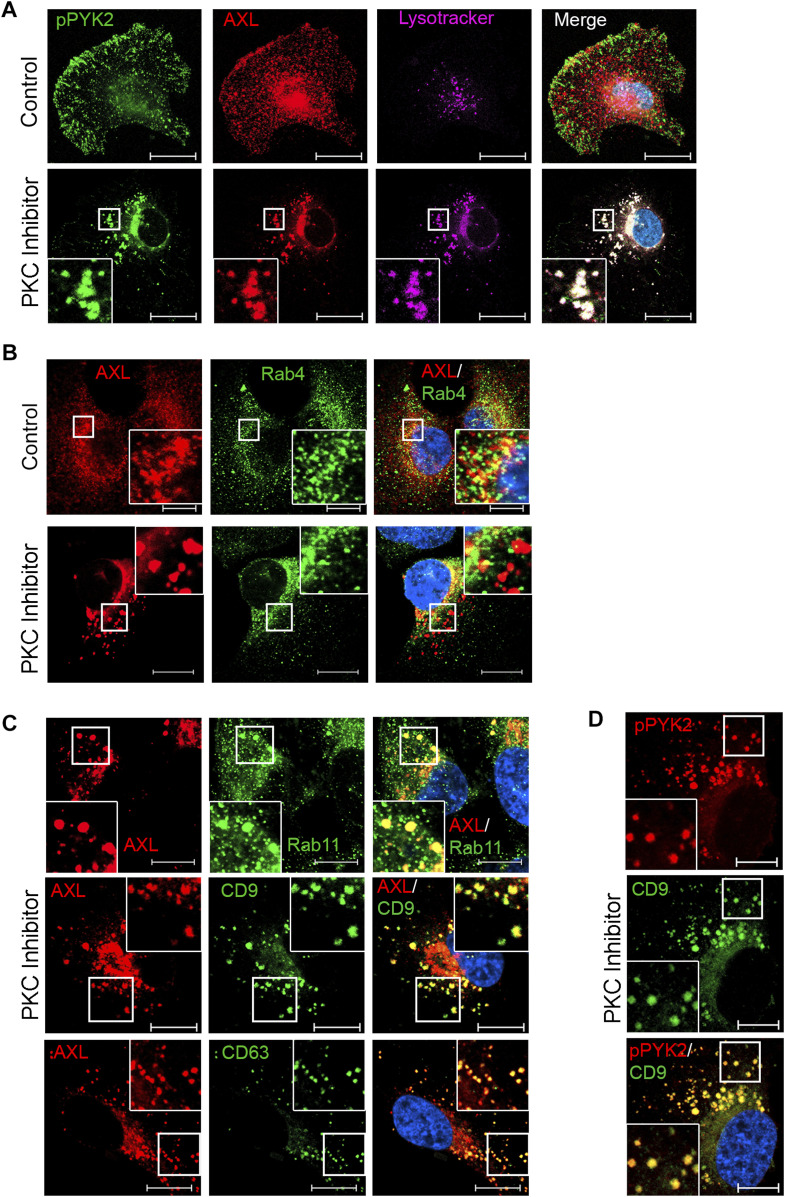
PKC inhibition induced redistribution of pPYK2 and AXL to endosomal/lysosomal compartment. The subcellular localization of pPYK2^Y402^ and AXL in control and PKC inhibitor (RO-31-8220)-treated (2 h) cells were assessed by IF analysis. Representative images of SUM159 cells are shown. Scale bar, 10 μm. Colocalization was estimated by the colocalization module of ZEN software (see the Materials and Methods section) and results are shown in Table S1. **(A)** Subcellular localization of AXL and pPYK2 with Lysotracker staining in control and RO-31-8220-treated SUM159 cells. **(B, C)** Subcellular localization of AXL (red) with the endosomal marker Rab4 (green) in control or RO-31-8220–treated SUM159 (B), or with the indicated endosomal/lysosomal markers (Rab11, CD9, and CD63) (green) in RO-31-8220–treated SUM159 (C). **(D)** Subcellular localization of pPYK2 (red) with CD9 (green) in RO-31-8220–treated SUM159. Scale bar, 10 μm.

**Figure S5.**
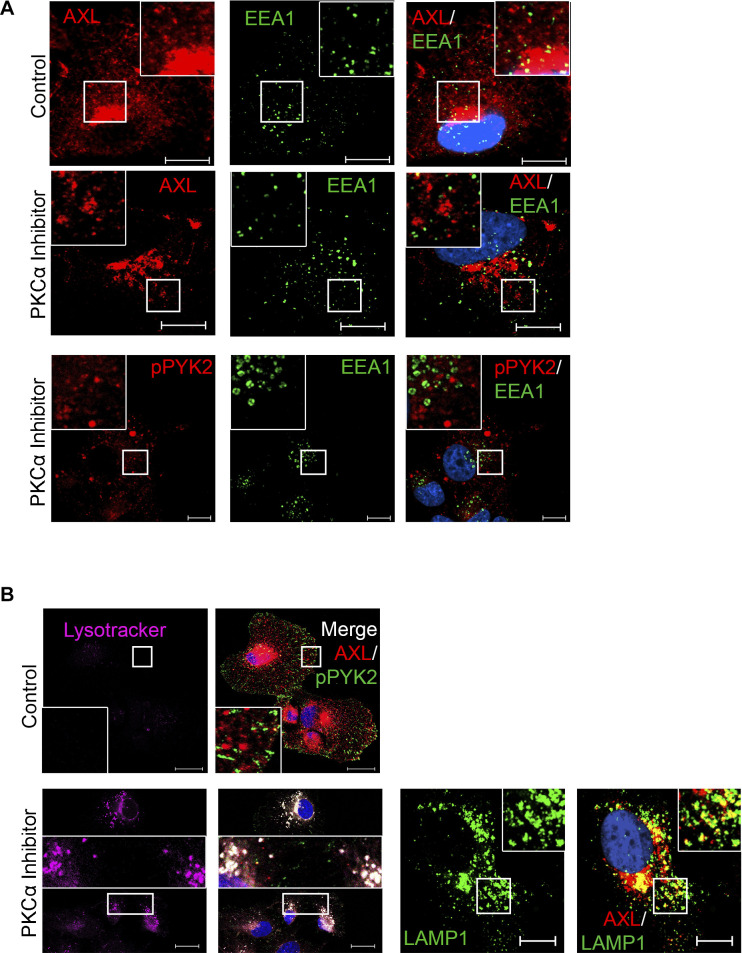
PKC inhibition enhanced lysotracker signal and induced redistribution of pPYK2 and AXL to lysosomes, distinctly from early endosomes. **(A)** The subcellular localization of the endosomal marker EEA1 (green) with either pPYK2^Y402^ or AXL (red) in control and PKC inhibitor (RO-31-8220)-treated (2 h) cells were assessed by IF analysis. Representative images of SUM159 cells are shown. Scale bar, 10 μm. Colocalization was estimated by the ZEN software (see the Materials and Methods section) and results are shown in Table S1. **(B)** Enhanced lysotracker signal and colocalization of AXL with lysotracker or LAMP1 upon PKC inhibition. Scale bar, 10 μm.

Whereas PYK2 depletion/inhibition reduced the transcription levels of AXL (Fig 1D and E), PKCα depletion/inhibition mainly affected the levels of AXL and PYK2 proteins (Figs 2A and S3D). To examine whether PKCα inhibition affects AXL degradation, RO-31-8220–treated cells were incubated with either MG132 or chloroquine. As seen, chloroquine treatment partially increased AXL and PYK2 levels in RO-31-8220–treated cells (Fig S4A), suggesting that inhibition of PKCα may facilitate the lysosomal degradation of AXL and PYK2. To further characterize the effect of PKCα on AXL and PYK2 fate, we examined their subcellular localization at different time points after PKCα inhibition. As shown in Fig 4A, short-term treatment for 2 h of SUM159 cells with the RO-31-8220 PKC inhibitor caused a redistribution of AXL and PYK2 from the cell-surface and focal adhesions, respectively, into punctate structures that were distributed throughout the cytosol, and largely colocalized with lysotracker. Similar effects were obtained with two additional PKC inhibitors (Gö6983 and GF109203X, Fig S4B). Localization studies using different cellular markers showed that PYK2- and AXL-positive punctate structures failed to colocalize with the early endosomal marker EEA1 (Fig S5A), marginally colocalized with the fast-recycling endosomal marker Rab4 (Fig 4B), but were strongly colocalized with Rab11, with the late endosomal/lysosome markers CD63 and CD9 (Fig 4B–D and Table S1), Lamp1 (Fig S5B) and with lysotracker (Fig 4A and Table S1). Strong colocalization with lysotracker was obtained with both AXL and pPYK2, concomitant with a significant increase in lysotracker signal intensity (Fig S5B) implying enhanced lysosomal activity (35). Colocalization with Rab11 was not observed with all Rab11-positive structures, but particularly with enlarged structures that likely represent late endosome/lysosome compartment (35), as Rab11 not only regulates recycling endosomes but also their fusion with multivesicular bodies (MVBs) (36), which can subsequently fuse with the lysosome. These results suggest that PKC inhibition impairs the endosomal–lysosomal pathway, and enhances rapid translocation of PYK2 to CD9-positive structures (Fig 4D) and lysosomal compartment (Figs 4A and S5B), where it was colocalized with AXL. These observations highlight a unique mode to control PYK2 and AXL levels and their downstream signals. Notably, PYK2/AXL–positive punctate structures could be captured at a narrow time window (2–4 h) using PKC inhibitors in the different M/MSL cell lines, but not in PKCα-depleted cells.

Table S1 Quantification of colocalization performed for each pair using Colocalization Module of ZEN software.

### The AXL-PYK2-PKCα axis correlates with stemness signatures in TNBC

**Figure 5.**
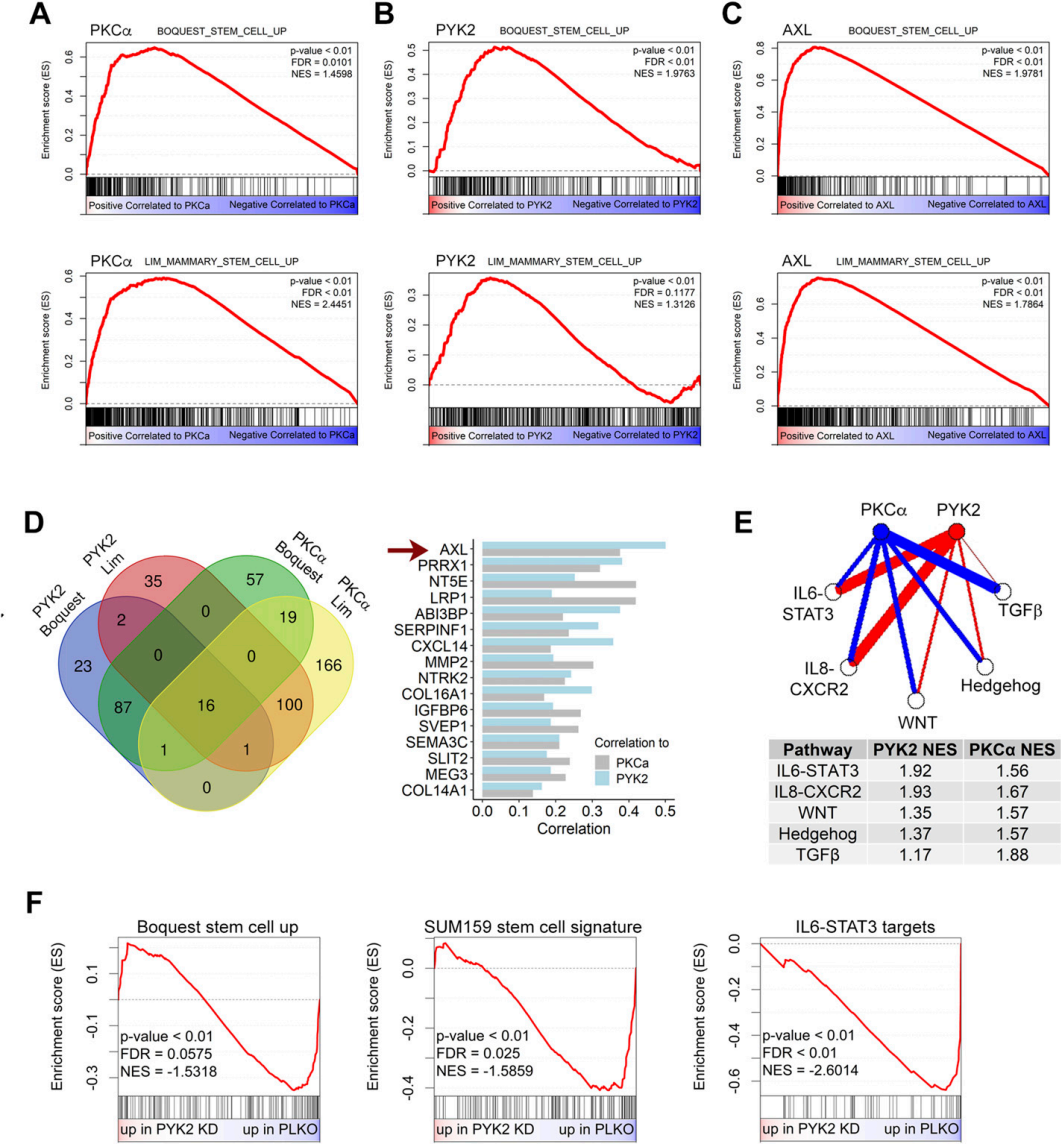
Gene set enrichment analysis (GSEA) of The Cancer Genome Atlas basal-like breast cancer (BC) patients in correlation to the AXL-PYK2-PKCα axis expression. **(A, B, C)** GSEA analysis of The Cancer Genome Atlas basal-like BC patients (n = 142). **(A, B, C)** For the analysis, all genes were ranked by their Pearson’s correlation to the gene expression of *PRKCA* (PKCα) (A), *PTK2B* (PYK2) (B), and *AXL* (C). The enrichment of two stemness signatures in these gene lists is shown and quantified as the normalized enrichment score (NES). **(A, B, D)** Leading edge analysis was performed on the GSEA results from (A) and (B). The leading-edge set is composed of genes from the signature that appear before the enrichment score peak is reached (genes that contribute the most to the enrichment score). **(A, B)** The Venn diagram (left) shows the intersections of the leading-edge sets in the four GSEA plots from (A) and (B). The 16 genes of the intersection from the four sets are shown in the plot on the right, with their correlations to PYK2 and PKCα gene expression. The genes are ordered by their correlation to PYK2 expression. **(E)** GSEA normalized enrichment scores (NES) for stemness-related pathways. Enrichment was measured in gene expression data of basal BC patients ranked by correlation to *PTK2B* (PYK2) or *PRKCA* (PKCα) expression. Line width represents the NES. **(F)** RNAseq results of PYK2 KD and control (PLKO) MDA-MB-231 cells were used to generate ranked list of genes based on fold change. Enrichment of the indicated signatures was performed using GSEA.

Previous studies showed that PKCα and its downstream effector FRA1 play key roles in driving CSCs of basal-like BC (27), implying that the AXL-PYK2-PKCα axis, which markedly affects FRA1 level/phosphorylation (Figs 2A and S3A and D), also regulates CSCs in TNBC. To assess the clinical relevance of this hypothesis, we first performed gene set enrichment analysis (GSEA) on basal-like BC patients of the TCGA dataset (n = 142), ranking the gene expression based to their correlation to *PRKCA* (PKCα) expression. This analysis revealed a significant positive enrichment of two stemness-related signatures (LIM_MAMMARY_STEM_CELL and BOQUEST_STEM_CELL_UP (4, 37, 38)) (Fig 5A). Similar results were also obtained for *PTK2B* (PYK2) (Fig 5B) and *AXL* (Fig 5C). Leading edge analysis of *PTK2B* and *PRKCA* enrichment plots (Fig 5A and B) revealed 16 common genes at the leading-edge sets of both signatures. Among these 16 genes, *AXL* appeared at the top, as the most correlated gene to *PTK2B* (PYK2), and the third highest correlated gene to *PRKCA* (Fig 5D). These results highlight the clinical relevance of the AXL-PYK2-PKCα axis to stemness in basal-like BC patients.

**Figure S6.**
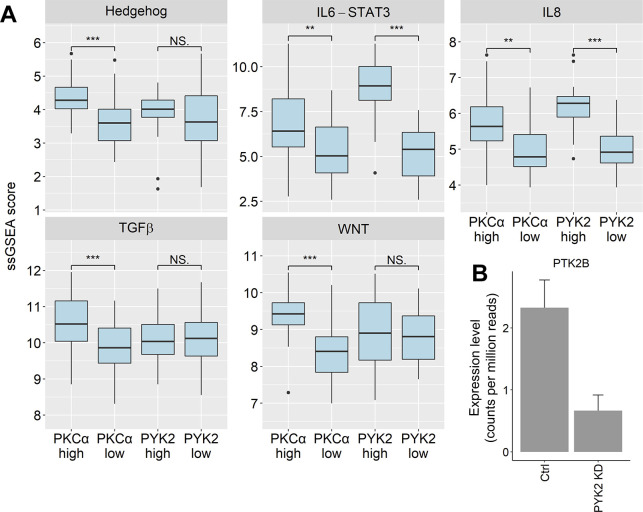
Stemness related pathways in basal breast cancer patients. **(A)** Single sample gene set enrichment analysis was used to score the enrichment of stemness related pathways in basal-like breast cancer patients from the The Cancer Genome Atlas dataset. Boxplots depicts the enrichment scores for the 20% highest and 20% lowest expressing PKCα or PYK2 patients (29 patients in each group out of 142 total). *P*-value (*t* test) ** < 0.01, *** < 0.001, NS, not significant. **(B)** Reads count of *PTK2B* gene from an RNAseq performed in MDA-MB-231 cells.

We next examined possible relation between PYK2 and PKCα expression and most prominent CSC-associated pathways, including the WNT, Hedgehog, TGFβ, IL6, and IL8 pathways (39). To this end, we scored each basal-like BC patient in the TCGA dataset for each of the five pathways using single sample gene set enrichment. We then compared the pathway scores in patients with high (top 20%) and low (bottom 20%) expression of PYK2 and PKCα (Fig S6A). Patients with low PYK2 expression had remarkable reduced scores of IL6-STAT3 and IL8 pathways, whereas patients with low expression of PKCα had reduced scores in all pathways, particularly, in Hedghehog, WNT and TGFβ pathways. This finding highlights the complementary effects of these two kinases on major stemness pathways in basal-like patients. To further corroborate our findings, we performed GSEA and examined the enrichment scores of the five pathways on gene lists ranked by correlation to either *PTK2B* or *PRKCA* expression (Fig 5E and Table S2). We observed similar relation between the two nodes, PYK2 and PKCα, and the stemness related pathways.

Table S2 Gene set enrichment analysis pathways used in Fig 5E.

To demonstrate the enrichment of stem cell signature in vitro, we analyzed PYK2 KD and control (PLKO) MDA-MB-231 cells by RNAseq (Fig S6B). We generated a ranked gene list, ordered by their fold change in expression between PYK2 KD and control (PLKO). Using GSEA on this list, we observed a negative enrichment score of the BOQUEST_STEM_CELL_UP signature that we have used for the patients above (Fig 5F, left), indicating that this signature is enriched in control (PLKO) and decreased in PYK2 KD cells, consistent with our dataset analysis. To strengthen this observation, in a more TNBC-related signature, we used a dataset of SUM159 cells sorted into stemness enriched population versus non-enriched population (GSE52262, (40)). The top 500 genes most significantly expressed in the stemness enriched population were highly expressed in control versus the PYK2 KD cells (Fig 5F, middle). Furthermore, we observed a significant negative enrichment of targets genes of IL6 signaling via STAT3 (Fig 5F, right). Together, these findings strongly suggest that the AXL-PYK2-PKCα axis is associated with CSCs signature of TNBC patients.

### The AXL-PYK2-PKCα axis regulates stemness in TNBC

**Figure 6.**
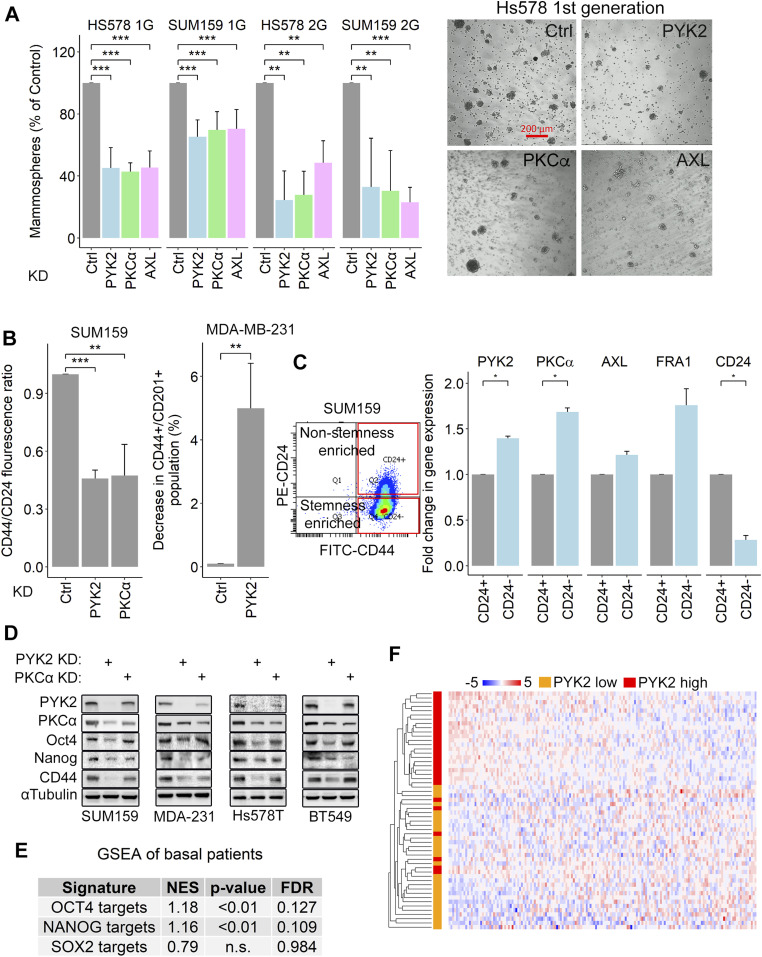
AXL-PYK2-PKCα axis regulates stemness in triple negative breast cancer. **(A)** Mammosphere formation assay was performed using SUM159 and Hs578T cell lines. The plot shows the percent of primary (1G) or secondary (2G) mammosphere formed (compared with control). Means ± SD of three independent repeats are shown. Representative images are shown to the right (Scale bar, 200 μm), and in Fig S7A. **(B)** FACS analysis of stemness surface markers. For SUM159, the CD44/CD24 fluorescence ratio is shown (Means ± SD of three independent repeats). For MDA-MB-231 the decrease in CD44^+^/CD201^+^ population is shown (Means ± SD of two independent repeats). Gating is shown in Fig S7B. *P*-value of KD versus control (*t* test)* < 0.05; ** < 0.01; *** < 0.001. **(C)** SUM159 cells were sorted by FACS into stemness enriched (CD44^+^/CD24^−^) and non-stemness enriched (CD44^+^/CD24^+^) populations. Gating for the sort is shown on the left. RNA was extracted and the gene expression was analyzed. Mean fold change in gene expression compared with the non-enriched (CD24^+^) population is shown from two experimental repeats. *P*-value of KD versus control (*t* test) * < 0.05. **(D)** The protein levels of the indicated stemness related proteins in control and PYK2 KD or PKCα KD triple negative breast cancer mesenchymal/mesenchymal stem–like cell lines were assessed by Western blot. **(E)** Gene set enrichment analysis of The Cancer Genome Atlas basal-like breast cancer patients. All genes were ranked based on their Pearson’s correlation to PYK2 expression. Enrichment of Oct4, Nanog, and Sox2 targets signatures was analyzed. “n.s.,” not significant. **(F)** Unsupervised clustering was performed using the expression levels of 166 targets of all three NOS (Nanog, Oct4, Sox2) transcription factors (taken from reference 48). Expression data were taken from basal-like breast cancer patients with the highest and lowest PYK2 expression in the The Cancer Genome Atlas (28 patients in each group).

**Figure S7.**
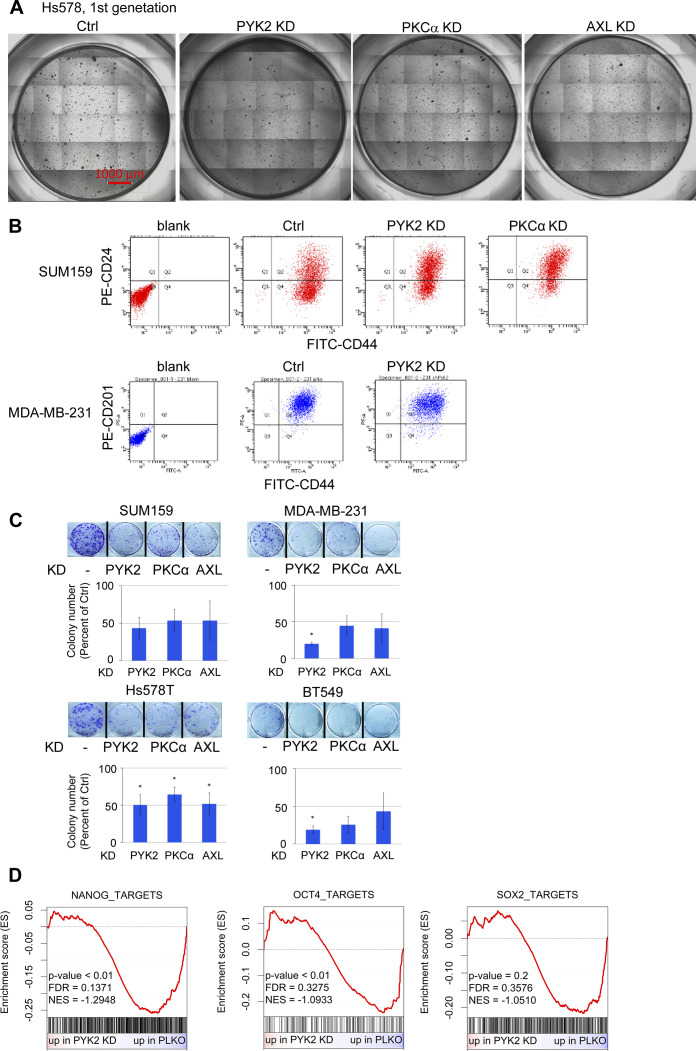
The AXL-PYK2-PKCα axis is involved in stemness in triple negative breast cancer. **(A)** Representative images of the Mammosphere formation assay. Images of the entire wells of 96-wells plate were taken by combining 7 × 7 images for each well. The diameter of the mammospheres was measured, and all mammospheres above 40 μm were quantified (plot shown in Fig 6A). Scale bar, 1,000 μm. **(B)** Representative FACS output for the results shown in Fig 6B. For SUM159 (top four plots) the fluorescence ratio between CD44 and CD24 was taken. For MDA-MB-231 (bottom three plots) the change in CD44^+^/CD201^+^ population was examined. **(C)** Representative pictures from colony formation assay performed using control and indicated KD cells. Bar graphs show mean percentage of number of colonies formed compared with control from two separate experiments. *P*-value of KD versus control (*t* test) *< 0.05. **(D)** RNAseq results of PYK2 KD and control (PLKO) MDA-MB-231 cells were used to generated ranked list of genes based on fold change. Enrichment of the indicated signatures was performed using gene set enrichment analysis.

To assess the phenotypic impact of the AXL-PYK2-PKCα axis on stemness, we performed a mammosphere formation assay as described in the Materials and Methods section. Hs578T and SUM159 were used as representative cell lines because of their established capability to generate mammospheres in vitro (41). As shown in Fig 6A, knocking down of PYK2, PKCα, or AXL markedly reduced the number of primary and secondary mammospheres compared with control cells (Figs 6A and S7A), highlighting their impact on maintaining the number of TICs and their self-renewal. We further showed by FACS analysis that PYK2 or PKCα depletion reduced the ratio of CD44 to CD24 levels on SUM159 cells surface (Figs 6B and S7B), and thus stem-cell enriched population (42). Similar trend was observed in PYK2-depleted MDA-MB-231 cells using the CD44^+^/CD201 (PROCR)^+^ ratio as a marker for stem-cell enriched population (43). Consistent with these results, we found that depletion of PYK2, PKCα, or AXL in the four M/MSL cell lines reduced the number of colonies in a colony formation assay for CSCs (44) (Fig S7C). Moreover, qRT-PCR analysis of PYK2, PKCα, and AXL transcripts in CD44^+^/CD24^−^ or CD44^+^/CD24^+^ sorted subpopulations of SUM159 cells, revealed their higher expression levels in the CD44^+^/CD24^−^ stemness enriched population (Fig 6C); collectively these results highlight the impact of the AXL-PYK2-PKCα axis on stemness in TNBC.

Key pluripotent TFs, including Nanog, Oct4, and Sox2 (NOS) are required for embryonic stem cell (ES) self‐renewal (45) and their target genes (NOS targets) are significantly enriched in CSCs of TNBC (46, 47). We, therefore, assessed the influence of PYK2 and PKCα depletion on their steady-state levels by WB. As shown in Fig 6D, knocking down of PYK2 reduced the levels of Oct4, Nanog and concomitantly CD44 in all the four M/MSL TNBC cell lines, without detectable effects on Sox2 (not shown), whereas PKCα knockdown reduced Nanog levels.

Concurrent with these observations, we found that the signatures of Oct4 and Nanog target genes (taken from reference 48) are significantly enriched in basal-like BC patients from the TCGA dataset when the genes were ranked by their correlation to PYK2 expression (Fig 6E). Similar results were observed in the RNAseq analysis performed on MDA-MB-231 PYK2 KD cells (Fig S7D). The expression levels of 166 targets of all three factors (Nanog, Oct4, and Sox2) clustered the PYK2 highly expressed basal-like BC patients separately from the PYK2 low-expressing patients (Fig 6F), demonstrating the clinical correlation between PYK2 expression and the activity of Nanog and Oct4 in those patients, and further highlighting the clinically relevance of our findings.

### TGFβ enhances stemness in TNBC and vulnerability to PYK2 and PKC inhibition

**Figure 7.**
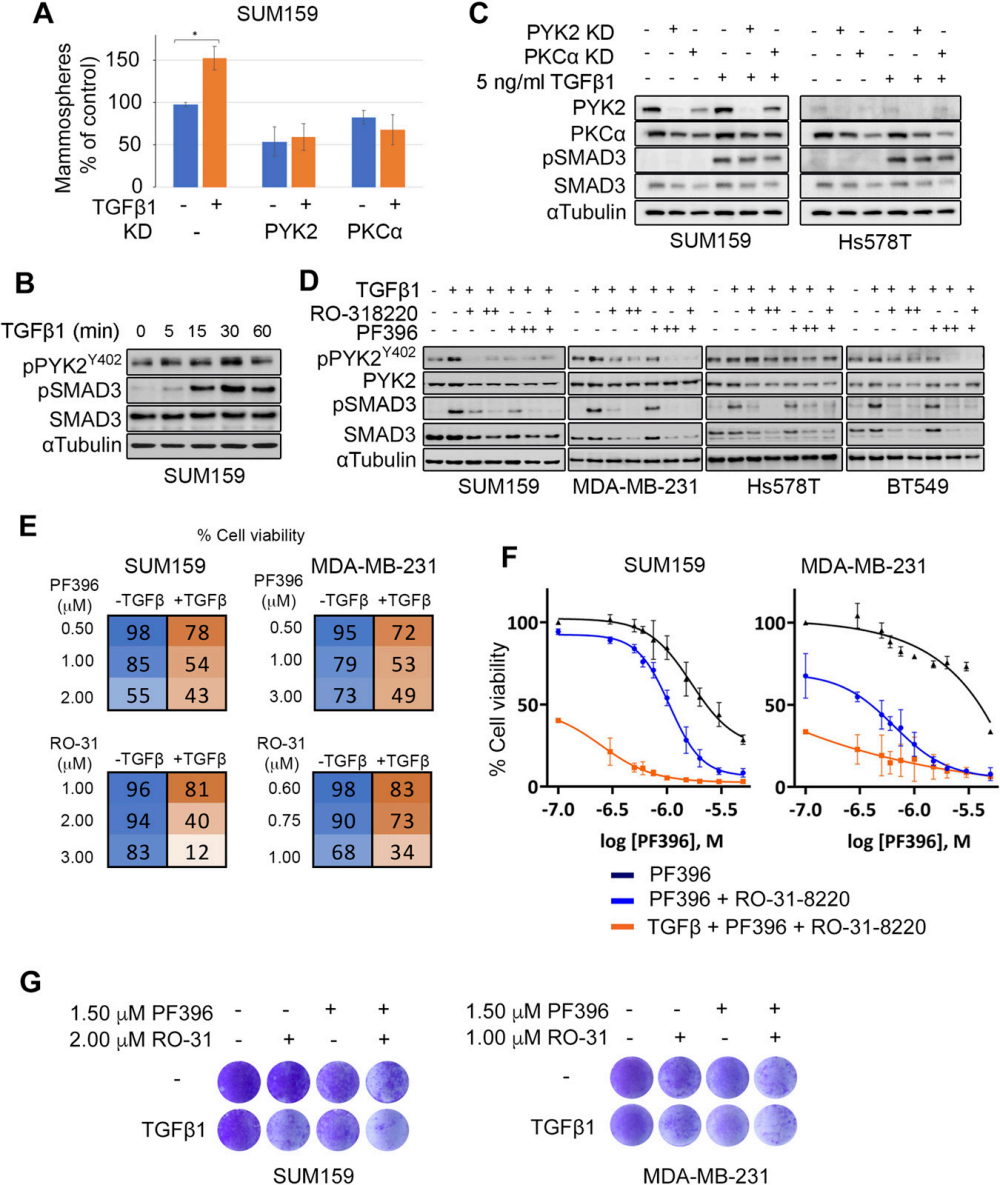
TGFβ enhances PYK2 phosphorylation, stemness, and vulnerability to PYK2 and PKC inhibition. **(A)** Control, PYK2-, or PKCα-KD SUM159 cells were treated with 2.5 ng/ml TGFβ1 for 72 h in full-growth media and then seeded for mammosphere formation assay. The plot shows the percent of primary mammospheres formed (compared with control). Means ± SD of two independent repeats are shown; *P-value of TGFβ1 treated versus control (*t* test) < 0.05. **(B)** SUM159 cells were stimulated with TGFβ1 (5 ng/ml) for the indicated time periods. Phosphorylation of PYK2(Y402) and SMAD3(S423/425) was examined by Western blot (WB). **(C)** Control, PYK2-, or PKCα-KD SUM159 and Hs578T cells were stimulated with TGFβ1(5 ng/ml) for 30 min. Phosphorylation of PYK2(Y402) and SMAD3(S423/425) was examined by WB. **(D)** The indicated mesenchymal/mesenchymal stem–like triple negative breast cancer cell lines were treated with PYK2 (PF396) or PKC (RO-31-8220) inhibitors overnight, and then serum starved for 2 h in presence of inhibitors before exposure to TGFβ1 (5 ng/ml for 30 min). PF396 was applied at 3 μM (+) or 6 μM (++), whereas RO-31-8220 was applied at 2 μM (+) or 4 μM (++) for SUM159, and 1 μM (+) or 2 μM (++) for the other three cell lines. Levels of PYK2, pPYK2 (Y402), SMAD3, and pSMAD3 (S423/425) were assessed by WB in the indicated cell lines. **(E, F, G)** The indicated cell lines were pre-treated with 10 ng/ml TGFβ1 for 24 h in full growth media. The medium was replaced with fresh media containing the indicated doses of PF396 or RO-31-8220, either alone or in combination. Cell viability was assessed 72 h later by MTT assay. **(E)** Cell viability was calculated relative to control untreated or TGFβ-treated cells and mean values of % cell viability from two independent experiments are shown for the indicated doses. Complete set of dose response ± SD is presented in Fig S8A. **(F)** Mean values of % cell viability for the indicated treatment were used to generate dose–response curves for PF396. RO-31-8220 was added at constant concentration of 2 μM for SUM159 and 1 μM for MDA-MB-231. **(G)** SUM159 or MDA-MB-231 cells were treated with the indicated concentration of PF396 and RO-31-8220 for 72 h with or without TGFβ pre-treatment, and then stained with crystal violet (see the Materials and Methods section). Shown are representative crystal violet staining results of two experiments.

Previous studies showed that TGFβ plays central role in EMT and cancer stemness (49) and enhanced stem-like properties of TNBC (19). Consistent with these reports, we found that pre-treatment of SUM159 cells with TGFβ for 72 h significantly increased the number of mammospheres as compared with naïve SUM159 cells (Fig 7A), and that knockdown of either PYK2 or PKCα reduced mammosphere numbers in both conditions (Fig 7A), implying that PYK2 and PKCα influence TGFβ-induced stemness signaling. To explore this possibility, we first examined whether PYK2 undergoes phosphorylation (pY402) in response to TGFβ stimulation (Fig 7B), and subsequently assessed the impact of PYK2 or PKCα depletion/inhibition on TGFβ downstream signals by monitoring SMAD3 phosphorylation (Fig 7C and D). As shown, TGFβ induced strong phosphorylation of its downstream effector SMAD3 as well as of PYK2 30 min after treatment (Fig 7B), and inhibition of either PYK2 or PKCα by PF396 or RO-318-220, respectively, markedly reduced SMAD3 phosphorylation, whereas their co-inhibition completely abolished phosphorylation of SMAD3 in the four examined TNBC cell lines (Fig 7D). Interestingly, the reduced phosphorylation of SMAD3 was associated with decrease in the steady-state level of SMAD3 protein. Knockdown of either PYK2 or PKCα also reduced pSMAD3 concomitant with total SMAD3 levels (Fig 7C), suggesting that PYK2 and PKCα cooperate downstream to TGFβ-receptor signaling.

**Figure S8.**
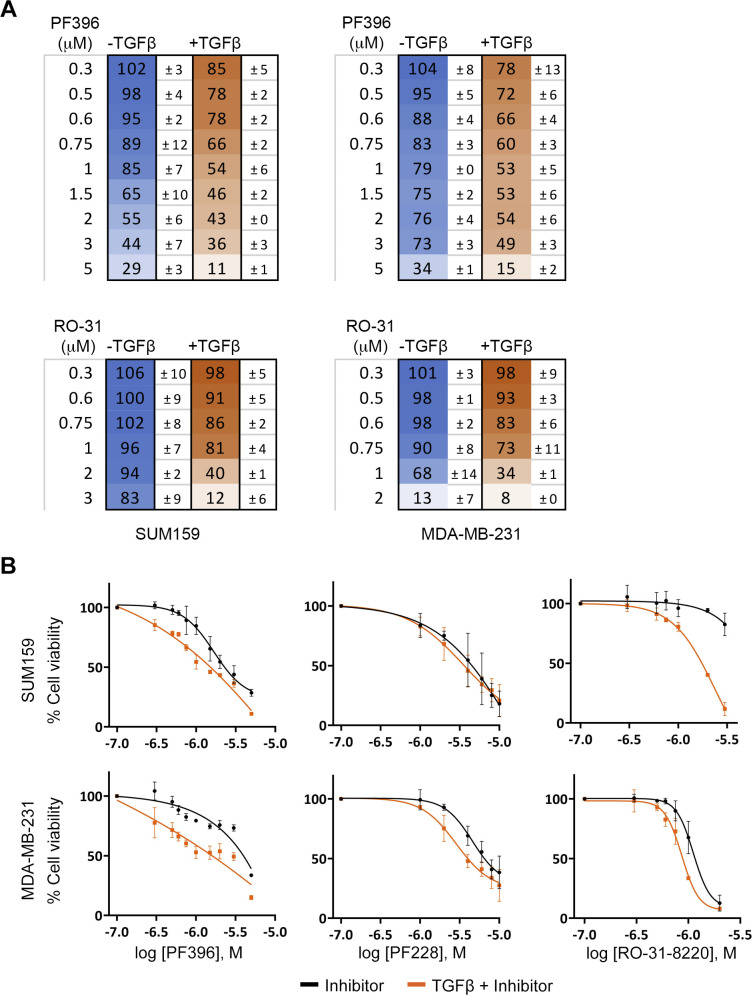
TGFβ enhanced vulnerability to PYK2 inhibition but not FAK inhibition. The indicated cell lines were exposed to 10 ng/ml TGFβ1 for 24 h, followed by treatment with indicated doses of PF396, PF228 or RO-31-8220 for further 72 h. MTT assay was performed and cell viability was calculated relative to control untreated or TGFβ-treated cells. **(A)** Mean values and SD of % cell viability from two independent experiments from Fig 7E and F. **(B)** Dose–response curves for indicated inhibitors and cell lines with or without TGFβ1 pretreatment.

In light of these results, we examined whether treatment with TGFβ, which enhances stemness (50), would increase vulnerability to PYK2 and/or PKCα inhibition. As shown, pre-treatment of either SUM159 or MDA-MB-231 with TGFβ, sensitized the cells to the PKC inhibitor RO-31-8220 or the PYK2/FAK dual-inhibitor PF396 (Figs 7E and G and S8A and B) but not to the FAK specific inhibitor PF228 (Fig S8B). Importantly co-inhibition of PKCα and PYK2/FAK substantially reduced cell viability in TGFβ-treated cells compared with TGFβ-untreated cells and displayed synergistic effect (combination index = 0.67 and 0.75 for SUM159 and MDA-MB-231, respectively, at 75% cell death) (Fig 7F), implying that stem cells could be more vulnerable to PKCα and PYK2 co-inhibition.

### Cooperation between PYK2 and PKCα modulates key stemness TFs

**Figure 8.**
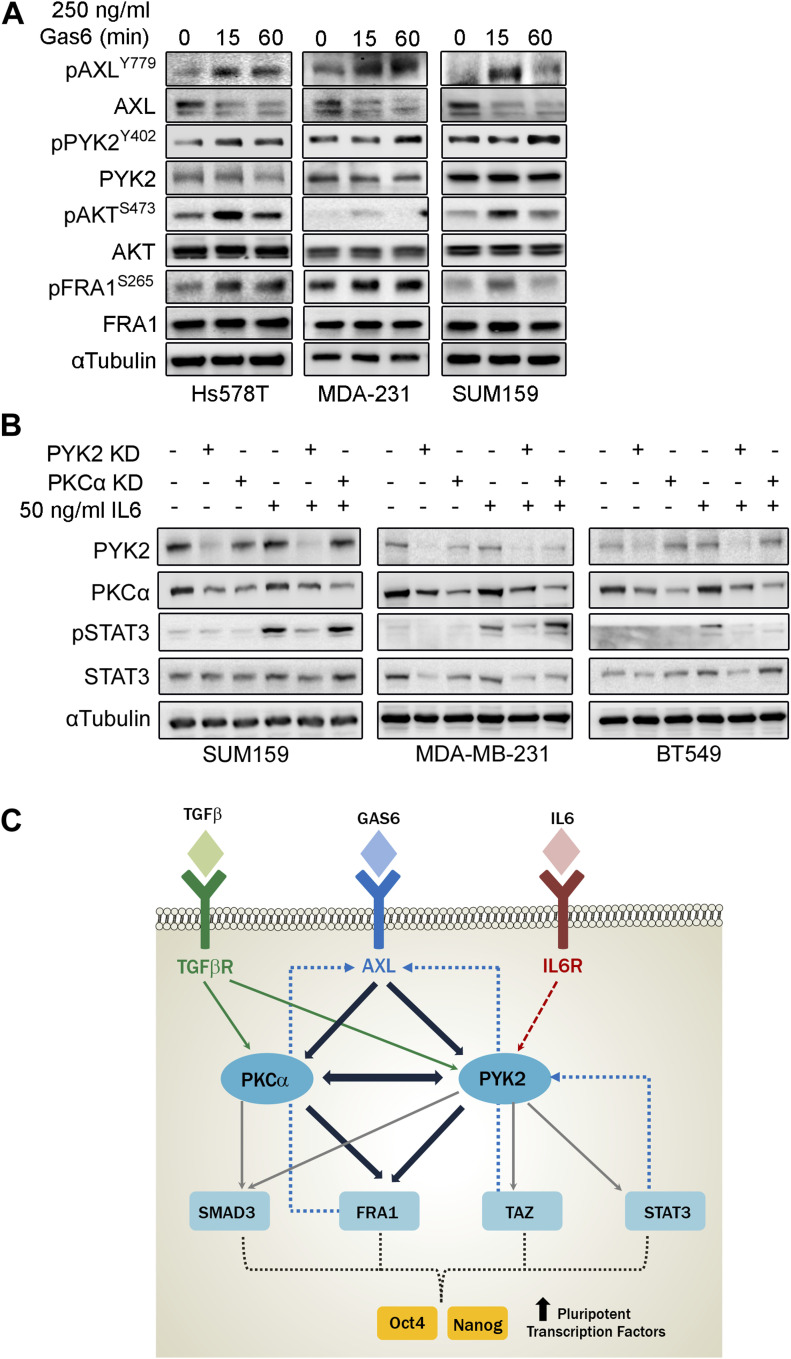
PYK2 and PKCα function at convergent point of multiple stemness-inducing pathways. **(A, B)** The different mesenchymal/mesenchymal stem–like triple negative breast cancer (TNBC) cell lines were serum starved for 2 h and then stimulated with either GAS6 (A) or IL6 (B). **(A)** The levels of total and phosphorylated AXL, PYK2, AKT, and FRA1 proteins in response to GAS6 (250 ng/ml) stimulation at the indicated time points was examined by Western blot. **(B)** Phosphorylation of STAT3 (Y702) in response to IL6 (50 ng/ml) stimulation for 30 min in control and PYK2 KD or PKCα KD TNBC mesenchymal/mesenchymal stem–like cells was examined by Western blot. **(C)** A scheme depicting the convergence of stemness promoting pathways at the PYK2 and PKCα signaling nodes. AXL-PYK2-PKCα axis acts at the center of stemness nexus in TNBC that modulates the level and/or activation of multiple transcription factors (TFs). PYK2 and PKCα are the central signaling nodes, converging signaling of different stemness promoting pathways, including TGFβ and IL6 pathways. TGFβ induces PYK2 and PKCα activation (green arrows), whereas PYK2 is required for STAT3 phosphorylation in response to IL6 (red dash arrow). PYK2 and PKCα mutually influence each other, and complementarily modulate the expression/activation of key TFs, including SMAD3, FRA1, TAZ, and STAT3, through phosphorylation, stabilization, and feedback loop mechanisms (blue dash arrows), thereby effectively regulating Nanog and Oct4 pluripotent TFs and stemness phenotype (the three receptors: TGFβR, AXL, and IL6R are illustrated in a schematic simplistic manner without structural details).

**Figure S9.**
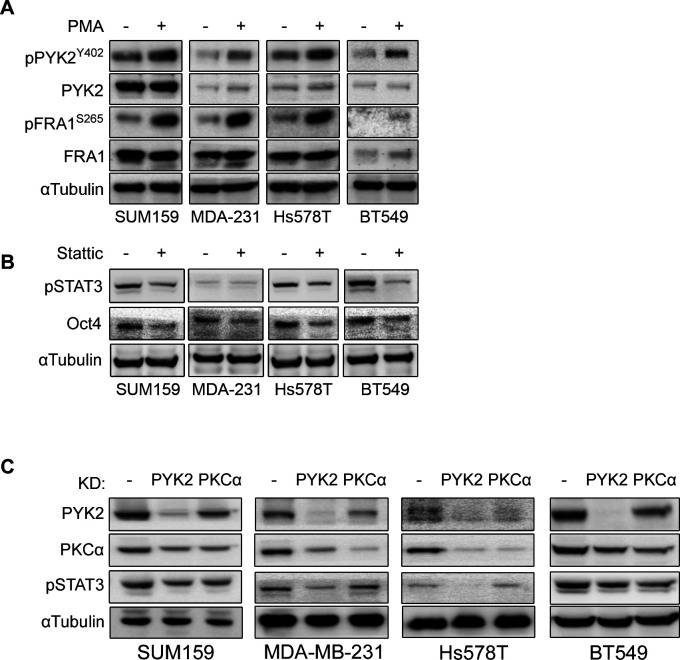
PKCα activation affects PYK2 and FRA1 phosphorylation, whereas PYK2 KD affects STAT3 phosphorylation and STAT3 influences Oct4 expression. **(A)** The indicated mesenchymal/mesenchymal stem–like triple negative breast cancer cell lines were serum starved for 2 h and then treated with PMA (10 ng/ml) for 1 h. Cell lysates were assessed for PYK2(Y402) and FRA1(S265) phosphorylation by Western blot (WB). **(B)** The indicated cell lines were treated with the STAT3 inhibitor Stattic (5 μM) for 8 h. Controls were treated with DMSO. The levels or phosphorylation of the indicated proteins were assessed by WB. **(C)** The levels of the indicated proteins in control, PYK2 KD, or PKCα KD triple negative breast cancer (mesenchymal/mesenchymal stem–like) cell lines were assessed by WB.

The remarkable effects of PYK2 and PKCα on the stemness properties of TNBC cell lines (Fig 6), and their established role as downstream effectors of multiple signaling pathways (51), suggest that these two kinases function as signaling nodes to converge stemness-related signaling pathways. As AXL activation induces EMT and is implicated in stemness (12), we first examined if AXL activation by its cognate ligand GAS6 induces phosphorylation of PYK2 as well as FRA1 in the four M/MSL TNBC cell lines. As seen (Fig 8A), GAS6 slightly enhanced the phosphorylation of PYK2 (pY402) and more strongly of FRA1 (pS265) and AKT (pS473), an established downstream effector of AXL signaling (30). Importantly, pPYK2 and pFRA1 were also observed upon PKC activation by PMA (Fig S9A), consistent with previous reports (29).

Previously, we showed that PYK2 positively regulates STAT3 phosphorylation (11, 52), and further studies showed that STAT3 plays an important role in CSC formation in BC, is implicated in Oct4 and Nanog transcription activation and in BCSCs self-renewal (20, 21). Indeed, inhibition of STAT3 by Stattic reduced the expression of Oct4 protein in the four M/MS cell lines (Fig S9B). STAT3 is also required for conversion of non-stem cancer cells into cancer stem-like cells downstream to IL6 receptor (53). Therefore, we examined the influence of PYK2 or PKCα depletion/inhibition on pSTAT3 under steady-state conditions and in response to IL6 activation. As shown in Fig 8B, IL6 induced strong phosphorylation of STAT3 in three of four M/MS cell lines (SUM159, MDA-MB-231, and BT549), and STAT3 phosphorylation was abolished in PYK2-depleted but not in PKCα-depleted cells, suggesting that PYK2 is essential for IL6-induced phosphorylation of STAT3. Similar effects were obtained under steady-state conditions (Fig S9C), demonstrating the strong impact of PYK2 on pSTAT3. Collectively, we show that PKCα and PYK2 affect AXL level and concomitantly critical signaling pathways and TFs that play central roles in regulating CSC in TNBC including FRA1, SMAD3, STAT3, and TAZ and consequently the transcription of pluripotent TFs Nanog and Oct4 and of stemness phenotype (Fig 8C).

## Discussion

Targeting of CSCs has been considered as a promising therapeutic approach for human cancer, in particular to recurrent, metastatic, and drug-resistant diseases (18). The major challenges are to identify this minor subpopulation of cancer cells, and eventually the specific molecular targets that eliminate CSCs.

Since the discovery of CSCs in the early 1990s, numerous studies have characterized their unique physiological features of slow proliferation rate, plasticity, self-renewal, and tumor-initiation capacity and concurrently identified discrete cellular markers, many of them are cancer type specific that have been used to isolate CSCs and profiling their transcriptome, proteome, and metabolome (39, 54).

These omics approaches yield stemness-associated transcriptomic signatures, such as the Lim mammary stem cell signature (38) (Fig 5) and uncovered stemness-associated signaling pathways that are crucial for CSCs survival and self-renewal. Targeting of these CSC-associated pathways has been used as a therapeutic strategy in many preclinical and clinical trials (55). In TNBC, several key pathways have been associated with stemness and proposed to be potent therapeutic targets, including the STAT3/Jak pathway, Wnt/β-Catenin, Notch pathway, Hedgehog, TFGβ, and AXL/GAS6 (3, 6). Importantly, many of these pathways crosstalk with each other, share downstream effectors, and are regulated by common feedback loops, implying that targeting of signaling nodes converging multiple stemness pathways could be an efficient strategy to simultaneously impair several CSC-related pathways and eliminate CSCs with high efficacy.

Here, we identified two signaling nodes, PYK2 and PKCα, that act at the convergent point of several stemness-associated pathways (Figs 5E, 7B and D, and 8), and functionally cooperate to regulate the pluripotent TFs Oct4 and Nanog and consequently stemness in TNBC (Fig 6D).

Previous studies suggested that activation of PKCα by PDGFR is crucial for switching non-CSCs to CSCs in TNBC, and that FRA1 downstream of PKCα regulates the expression of EMT-CSC program (27). Here, we show that PKCα expression is strongly influenced by PYK2 (Fig 1D), that PYK2 is a downstream signaling component of additional key stemness-inducing pathways, including TGFβ and GAS6/AXL (Figs 7 and 8), and that PKCα and PYK2 mutually regulate each other and cooperate to robustly modulate stemness in TNBC (Fig 6). Consistent with their mode of action, our attempt to simultaneously deplete PYK2 and PKCα in M/MSL TNBC cell lines repeatedly failed, and the few cells that survived grew extremely slow (not shown). Nevertheless, we showed that inhibition of their kinase activities using small molecule inhibitors markedly reduced the viability of TGFβ-treated TNBC cells and their co-inhibition had synergistic effects (Fig 7E and F). These results suggest that targeting of PYK2 and PKCα could effectively eliminate CSC-enriched population. Interestingly, we previously showed that co-targeting of PYK2 and EGFR could be beneficial for basal-like patients with high expression of EGFR (52), and here we propose that co-targeting of PYK2 and PKCα might be more effective for EMT/stemness-associated tumors.

The cooperation between PYK2 and PKCα is also reflected by their complementary influence on AXL levels (Figs 1 and 2); whereas PYK2 depletion/inhibition markedly reduced the protein and transcription levels of AXL, PKCα inhibition mainly affected the protein level of AXL, induced its translocation into an endosomal/lysosomal compartment that was positive for Rab11 and for the MVBs/late endosomal markers CD63/CD9, Lamp1, and lysotracker, and enhanced its lysosomal degradation (Figs 4 and S4). These results imply that PKC inhibition perturbs the endosomal–lysosomal pathway. Although future studies will be required to uncover the underlying mechanism, it is possible that PKC inhibition impairs Rab11-associated functions, as Rab11 was reported to be phosphorylated by PKC (56), and Rab11 depletion induced punctate structures resembling the structures obtained by PKC inhibition (Figs 4A and S4B), and also increased lysotracker staining (35). Importantly, PYK2 was also localized to these structures and colocalized with AXL, demonstrating the mutual interplay between components of the AXL-PYK2-PKCα axis.

The robust effect of PYK2 on AXL was observed in all the M/MSL TNBC and also in other cell types (not shown), further supporting the link between these two kinases (Fig 1D). This was also reflected by dataset analysis of TNBC and the finding that AXL was the top gene among the 16 stemness correlated genes with PYK2 and PKCα stemness signatures (Fig 5D). The substantial effect of PYK2 on AXL is mediated, at least in part, by feedback loop of two transcription regulators, the transcription co-activator TAZ (34) and TF FRA1 (Fig 3). This finding has an important clinical implication as it strongly suggests that inhibition of PYK2 could overcome AXL-associated drug resistance, which is frequently associated with resistance to different anticancer drugs, including chemotherapy (13). Moreover, PYK2 and AXL are highly expressed in immune cells and possibly exhibit a similar mutual influence.

Identification of PYK2 and PKCα as clinically relevant signaling nodes of different stemness-associated pathways (Figs 7 and 8) is reflected by their combined effects on multiple TFs such as STAT3, TAZ, FRA1, and SMAD3 (Figs 2A, 3C, 7C, and S9B) and the subsequent pluripotent TFs Oct4 and Nanog (Fig 6D). Importantly, we have previously showed that PYK2 regulates STAT3 during EMT in TNBC and that STAT3 via a feedback loop regulates PYK2 expression (52, 57). These results highlight how this stemness nexus is convergent and controlled by different feedback loops that together ensure the robustness of stemness phenotype. We, therefore, propose that combined targeting of PYK2 and PKCα could be beneficial for CSCs elimination in TNBC and possibly overcoming drug resistance.

## Materials and Methods

### Antibodies, reagents, and chemicals

Antibodies to AXL (sc-166268), FRA1 (sc-605), PKCα (sc-208), pPYK2 (Y402, sc-101790), FAK (sc-932), pSTAT3 (sc-8059), STAT3 (sc-483), YAP/TAZ (63.7, sc-101199), Rab11 (sc-9020), and PKC inhibitor RO-31-8220 (sc-200619) were purchased from Santa Cruz Biotechnology. Antibodies to pSMAD3 (9520), SMAD3 (9523), pFAK (8556), pFRA1 (5841), Oct4 (2750S), and Nanog (3580S) were purchased from Cell Signaling Technologies. Antibodies to CD44 FITC (BY18, 338804) and CD24 APC (ML5, 311118) were purchased from BioLegend. Antibody to α-tubulin (T6074) as well as the following chemicals, Hoechst 33342, Verteporfin (1711461), PF431396 (PZ0185), and Chloroquine (C6628) were purchased from Sigma-Aldrich Israel. Gö6983 (S2911) and GF109203X (S7208) were from Selleck chemicals. Polyclonal anti-PYK2 antibody was prepared as described previously (58). Antibody to CD44 was from Hybridoma clone H4C4. Antibody to Rab4 (ab13252; Abcam) was kindly provided by Prof. B. Aroeti (HUJI). Cyanine Cy3–conjugated goat antirabbit and goat antimouse immunoglobulin Gs (IgGs) were purchased from Jackson ImmunoResearch Laboratories. Alexa-488 donkey antimouse and antirabbit IgGs were purchased from Invitrogen. PF573228 (324878) and MG132 (474790) were purchased from Calbiochem. LysoTracker Red DND-99 was purchased from Thermo Fisher Scientific. TGFβ1 was purchased from ProspecBio. Human IL6 was from Genscript (Z03034).

### Cell culture

All cell lines that were used in the study were originally obtained from ATCC. HCC70, MDA-MB-468, MDA-MB-231, BT549, SUM159, and Hs578T were maintained in RPMI (Gibco BRL). HEK293 cells were maintained in DMEM (Gibco BRL). Media were supplemented with 10% vol/vol fetal bovine serum (Gibco BRL) and 1% vol/vol penicillin–streptomycin mixture (Beit Haemek) unless otherwise stated. Cell lines were regularly verified to be mycoplasma negative.

### Immunoblot analysis

Cells were washed with cold PBS and lysed in cold lysis buffer (0.5% Triton-X-100, 50 mM Hepes pH 7.5, 100 mM NaCl, 1 mM MgCl_2_, 50 mM NaF, 0.5 mM NaVO_3_, 20 mM β-glycerolphosphate, 1 mM phenylmethylsulphonyl fluoride, 10 μg/ml leupeptin, and 10 μg/ml aprotinin), vortexed, and incubated on ice for 30 min with vortexing at intervals of 10 min. Cleared cell extracts were obtained by centrifuging at 19,300*g* for 15 min at 4°C. Bradford assay (Bio-Rad) was used to estimate sample protein concentrations and equal amounts of total protein per sample were analyzed on SDS–polyacrylamide gel electrophoresis and Western Transfer using standard procedures. A 5% nonfat dry milk solution in TBS-Tween (0.05%) was used for blocking at room temperature for 1 h. Incubation with primary antibodies was performed at 4°C overnight on a rocker. Incubation with secondary antibodies was performed at room temperature for 1 h. For densitometric analysis, the intensity of protein bands was measured using the FIJI-ImageJ software (NIH). The band intensities of analyzed proteins of WBs were normalized to that of α-tubulin.

### RNA extraction and real-time PCR analysis

RNA was purified using TRI Reagent (T9424; Sigma-Aldrich). cDNA was generated using High-Capacity cDNA Reverse Transcription Kit (Applied Biosystems; Invitrogen). Real-time PCR analysis was performed using SYBR Green as a fluorescent dye, according to the manufacturer’s guidelines using the ABI StepOnePlus 7500 Real-time PCR system (Applied Biosystems; Invitrogen). All experiments were normalized to Actin RNA levels. The primer sequences are provided in Table S3.

Table S3 List of qRT-PCR primers.

### Immunofluorescence staining

Cells were grown and treated on sterile coverslips placed in wells of a 24-well plate, washed with PBS at 35°C, and fixed in 4% paraformaldehyde solution, also preheated at 35°C, for 15–20 min at room temperature. The fixed cells were then washed once with room temperature PBS and incubated for 10 min in PBS containing 0.1 M glycine to quench excess PFA action. This was followed by blocking with TBS containing 0.1% Triton-X-100, 10% goat serum, and 2% BSA for 30 min. Incubation with the primary antibody was carried out at room temperature for 1–2 h, followed by three washes in PBS, and then 1 h incubation with the secondary antibody. The cells were incubated for 5 min with PBS containing 2 μg/ml Hoechst 33342, washed three times with PBS, and mounted on microscopic slides using mounting media (10 mM phosphate buffer, pH 8.0, 16.6% wt/vol Mowiol4–88, and 33% glycerol). The prepared slides were analyzed by using Zeiss LSM800 confocal laser-scanning microscope. Colocalization was calculated using Colocalization Module of ZEN software (Zeiss Microscopy LLC). Pearson’s coefficient and colocalization coefficient for each channel are presented in Table S1.

### shRNA lentivirus–mediated knockdown

PYK2 expression was down-regulated by two different shRNAs: shRNA #519 (TRCN00000231519) was purchased from Sigma-Aldrich, whereas the second one (shRNA #5) was prepared as previously described (59). Both shRNA showed similar effects. Experiments shown in the manuscript were performed with shRNA #519. AXL expression was down-regulated by two different shRNAs: shRNA #971 (TRCN0000194971) was kindly provided by Prof. M Elkabets, whereas shRNA #699 (TRCN0000001038) was kindly provided by Dr. D Lin. Both shRNA showed similar effects. Experiments shown in the manuscript were performed with shRNA #971. PKCα expression was down-regulated by three different shRNAs: shRNA #690 (TRCN0000001690) was prepared by cloning into pLKO.1-puro lentiviral vector, shRNA #691 (TRCN0000001691), and shRNA #692 (TRCN0000001692) were kindly provided by Prof. RS Harris. The different shRNA showed similar effects. Experiments shown in the manuscript were performed with shRNA #690. FRA1 expression was down-regulated using shRNA sequence TRCN0000019539. The respective shRNA sequences were cloned into the pLKO.1-puro lentiviral vector. Selection medium for infected cells contained added puromycin at 1.5–2 μg/ml.

### Cell viability assay

Cells were plated in 96-well plates and next day treated with TGFβ1 (10 ng/ml) for 24 h. The cells were then treated with the indicated concentrations of PYK2/FAK (PF396), FAK (PF228), or PKC (RO-31-8220) inhibitors, either alone or in combination in fresh media using DMSO as vehicle control. Inhibitor-treatment was continued for 72 h and cell viability was measured by MTT assay (60). Cell viability is shown as percentage of control. Synergy of PYK2 and PKCα inhibitors was calculated by the CompuSyn software using the Chou–Talalay equation. Combination index is given for the concentration of inhibitors that induces a combined effect of 75% cell death.

### Dataset analysis

Enrichment of AXL in MSL subtype patients was shown in datasets of TNBC patients taken from Lehmann et al, (28). For subsequent analysis, gene expression data of TNBC patients were taken from TCGA dataset (n = 142 basal-like BC patients). Cell lines data were taken from the Cancer Cell Line Encyclopdia dataset (n = 54 BC cell lines). GSEA were performed using GSEA software (Broad institute). Single sample gene set enrichment was performed using GenePattern (www.genepattern.org/). All other dataset analysis was performed in R.

### RNAseq

RNA from PYK2 KD and control MDA-MB-231 cells was extracted by TRIzol as described above and used to generate RNAseq libraries applying a bulk adaptation of the MARS‐seq protocol, as described previously (61). Libraries were sequenced by the Illumina Novaseq 6000 using SP mode 100 cycles kit (Illumina). Mapping of sequences to the genome was performed by the user-friendly transcriptome analysis pipeline (Weizmann Institute) (62). Library normalization, filtration of low count genes, and discovery of differentially expressing genes was performed using the edgeR package in R. For GSEA, genes were pre-ranked by their fold change in PYK2 KD versus control cells. RNAseq was performed in two biological replicates.

### Mammosphere assay

Generation of mammospheres for detection of TICs was performed as described previously (63). In brief, single-cell suspensions were prepared by trypsinization and disengagement using a 25G needle syringe. Cells were seeded at low density (10,000 cells/cm^2^) in poly-hema–coated six-well plates (poly-hema was dissolved in 95% ethanol, 20 mg/ml). Plates were incubated with the poly-hema over-night in RT. Cells were grown in mammosphere medium (DMEM/F12 medium, without serum, supplemented with 2 mM L-glutamine, and 100 U/ml penicillin/streptomycin, 20 ng/ml EGF, 10 ng/ml βFGF and B27 1:50 [Thermo Fisher Scientific]). After 10 d of incubation, the medium was centrifuged (150*g* for 5 min), the mammospheres were resuspended in 200 μl PBS and placed in a well of a 96-well plate. The entire well was photographed and the diameter of all mammospheres was measured using ZEN lite software (Zeiss). Mammosphere with a diameter above 40 μm were counted. Alternatively, after 10 d in culture, mammospheres were passaged (by trypsinization and a 25-G needle, seeded in the same density as before), to generate secondary mammospheres which were incubated and quantified in a similar manner.

### Colony formation assay

Control or KD cells were prepared in single cell suspension. 1,000 cells/well were plated in six well plates in duplicates and allowed to grow for 7–10 d until colonies were visible under the microscope. Colonies formed were stained with Crystal Violet, photographed, and counted.

### FACS assay

The surface expression of stemness markers (CD44 and CD24 for SUM159, CD44 and CD201 [PROCR] for MDA-MB-231) was carried out as follows. Cells were trypsinized briefly and washed with PBS, filtered using a 40-μm mesh, and collected in Eppendorf tubes, 1 × 10^6^ cells/tube. The cells were incubated with the antibodies at a dilution of 1:200 in FACS buffer (3% FBS in PBS) for 20 min, 4°C in the dark. After incubation, the cells were washed twice in PBS. The cells were analyzed using SORP-LSRII flow cytometer (BD Biosciences). For sorting SUM159 cells into CD44^+^/CD24^+^ and CD44^+^/CD24^−^ populations, the cells were prepared as above and sorted using FACSAria II flow cytometer (BD Biosciences). RNA was extracted from each population by Trizol, and qRT-PCR was performed as described above. Antibodies used for FACS analysis were purchased from BioLegend (CA): FITC-CD44 (Cat. no. 338804), APC-CD24 (Cat. no. 311118), and PE-CD201 (Cat. no. 351903).

### Statistical analysis

Two-tailed independent *t* test was performed to analyze the results of all assays. Error bars in figures represent SD of the experimental repeats.

## Data Availability

The RNAseq data from publication has been deposited to the GEO database (https://www.ncbi.nlm.nih.gov/geo/) and assigned the accession number GSE166609.

## Supplementary Material

Reviewer comments
