# Supplementary figures and images for: The AXL-PYK2-PKCα axis as a nexus of stemness circuits in TNBC
Source: Life Sci Alliance. 2021 Mar 30;4(6):e202000985. doi: 10.26508/lsa.202000985 (PMC8046419; doi:10.26508/lsa.202000985)

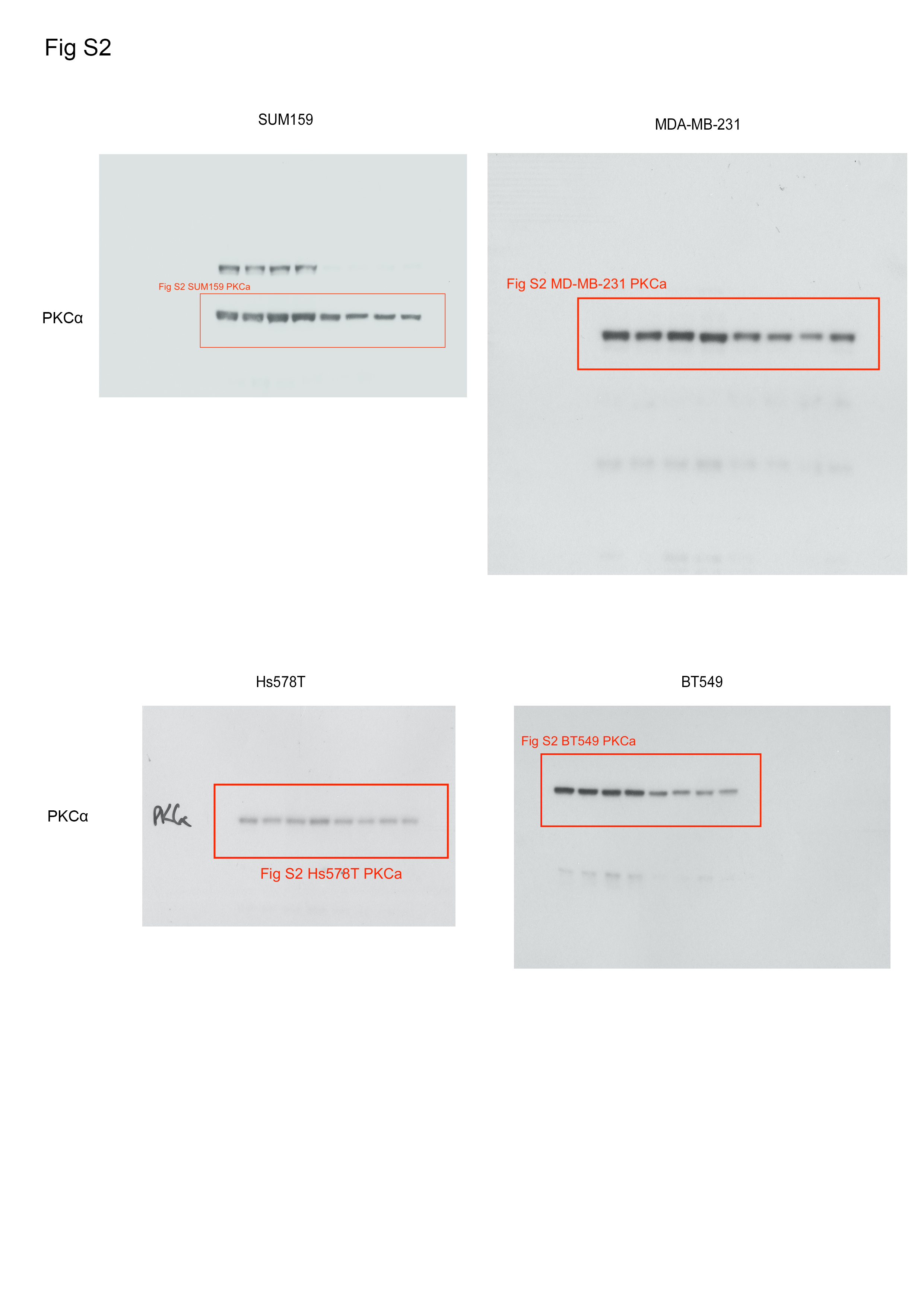

Supplement: Supplementary file 1 [file LSA-2020-00985_SdataFS2.tif]
